# What About Their Performance Do Free Jazz Improvisers Agree Upon? A Case Study

**DOI:** 10.3389/fpsyg.2017.00966

**Published:** 2017-06-26

**Authors:** Amandine Pras, Michael F. Schober, Neta Spiro

**Affiliations:** ^1^Department of Psychology, New School for Social Research, The New SchoolNew York, NY, United States; ^2^Department of Music, University of LethbridgeLethbridge, AB, Canada; ^3^Research, Nordoff Robbins Music TherapyLondon, United Kingdom; ^4^Centre for Music and Science, Faculty of Music, University of CambridgeCambridge, United Kingdom

**Keywords:** free jazz, improvisation, shared understanding, music cognition, intersubjectivity, creative process, collaboration, interrater agreement

## Abstract

When musicians improvise freely together—not following any sort of script, predetermined harmonic structure, or “referent”—to what extent do they understand what they are doing in the same way as each other? And to what extent is their understanding privileged relative to outside listeners with similar levels of performing experience in free improvisation? In this exploratory case study, a saxophonist and a pianist of international renown who knew each other's work but who had never performed together before were recorded while improvising freely for 40 min. Immediately afterwards the performers were interviewed separately about the just-completed improvisation, first from memory and then while listening to two 5 min excerpts of the recording in order to prompt specific and detailed commentary. Two commenting listeners from the same performance community (a saxophonist and drummer) listened to, and were interviewed about, these excerpts. Some months later, all four participants rated the extent to which they endorsed 302 statements that had been extracted from the four interviews and anonymized. The findings demonstrate that these free jazz improvisers characterized the improvisation quite differently, selecting different moments to comment about and with little overlap in the content of their characterizations. The performers were not more likely to endorse statements by their performing partner than by a commenting listener from the same performance community, and their patterns of agreement with each other (endorsing or dissenting with statements) across multiple ratings—their interrater reliability as measured with Cohen's kappa—was only moderate, and not consistently higher than their agreement with the commenting listeners. These performers were more likely to endorse statements about performers' thoughts and actions than statements about the music itself, and more likely to endorse evaluatively positive than negative statements. But these kinds of statements were polarizing; the performers were more likely to agree with each other in their ratings of statements about the music itself and negative statements. As in Schober and Spiro (2014), the evidence supports a view that fully shared understanding is not needed for joint improvisation by professional musicians in this genre and that performing partners can agree with an outside listener more than with each other.

## Introduction

When musicians improvise freely together—not following any sort of script, predetermined harmonic structure, or “referent” (Pressing, 1984)—to what extent do they understand what they are doing in the same way as each other? And to what extent is their understanding privileged relative to outside listeners with similar levels of performing experience in free improvisation?

This case study addresses these questions in the context of joint duo improvisation, where what one performer plans and executes is of necessity shaped by the other performer's simultaneous actions, and the direction of the joint creation can be different from what either performer individually would have chosen. In joint musical performance, as in many other kinds of joint action like conversation, dancing together, or even shaking hands (Clark, 1996), each performer's thoughts and actions are by definition interdependent; that is, each performer's cognitive and behavioral requirements go beyond the already-complex requirements of performing from a single performer's perspective to involve the *other* performer's decisions and responsiveness (e.g., Loehr et al., 2013; Keller, 2014, among many others). Joint musical *improvisation* has yet different dynamics; when each musician's improvisational process (see e.g., Sloboda, 1985; Pressing, 1988; Biasutti and Frezza, 2009; Donnay et al., 2014; Dean and Bailes, 2016) depends on less predictable moves by the other, the degrees of freedom increase. In our view, given the indeterminacy or “floating intentionality” of musical meaning more generally (Cross, 2014), what exactly performers share during joint improvisation is an open question.

Our focus is on highly skilled professional musicians playing in the domain of free improvisation, which prioritizes flexibility and unpredictability even beyond what is valued in the improvisation on jazz standards that is more frequently studied. We examine the extent to which two professional free jazz improvisers agree about what happened in their first free improvisation together, using the method from a previous case study of two professional musicians improvising on a jazz standard (Schober and Spiro, 2014). That study suggested that fully shared understanding of what happened may not be essential to jazz improvisation: performers endorsed statements made by a commenting listener more than they endorsed statements by their performing partner, and their overall level of agreement with each other's characterizations was not high. The question in this paper is whether this pattern is also observable in freer improvisation, and among first-time co-performers who have met before and have some prior familiarity with each other's playing.

### Free jazz improvisation

Free improvisation, commonly known as free jazz, has been defined as “non-idiomatic improvisation” (Bailey, 1993), in that the improvisation is not based on pre-established musical elements (rhythm, harmony, melody, or timbre), nor necessarily on esthetically pleasing ways of playing or phrasing. Berliner (1994) characterizes free jazz as an idiom that reflects “the ideological rejection of former jazz convention” (p. 122) and that pushes the boundaries of improvisational norms in a range of different ways. As Canonne and Garnier (2015, p. 1) put it, in this kind of improvisation musicians deliberately avoid the kinds of “shared frameworks” that allow more traditional improvisers to “collectively organize their individual contributions into a coherent temporal form.” Modern free jazz practice has multiple roots and offshoots (Arthurs, 2015a,b), and is traced in Lewis' (1996) formulation to two significant post-war traditions of non-idiomatic improvisation: an “Afrological” approach that arises from the political and historical dimensions of African-American music (a tradition that Lewis sees as including Charlie Parker, Cecil Taylor, John Coltrane, and Ornette Coleman), and a “Eurological” approach rooted in European classical and contemporary music traditions (emblematically John Cage and Morton Feldman).

As demonstrated in Pras' (2015a,b) semi-directed interviews with 12 New York-based professional touring improvisers (all from different geographical, cultural, musical, and instrumental backgrounds but within the same local performance community), different practitioners can have different preferences for what the genre should be called, as well as different ideas about what it encompasses and what is prioritized. For example, while some performers reject the idea that free jazz is an idiom at all, pianist Matthew Shipp claimed that over time, free jazz had become an idiom, with “a few gestural things that performers pick up on pretty closely” (Pras, 2015a). Several improvisers rejected the “free jazz” label as not reflecting their values; drummer Todd Capp mentioned that “free jazz” has in the past sometimes been associated with “loud aggressive egocentric macho music” (Pras, 2015a). These are plausible reasons for why many improvisers prefer using “free improvisation” or just “jazz,” or alternatively “spontaneous composition.” Here we will use “free jazz improvisation” because it seems the most general in the community of artists that we are working with, though we recognize that the terms are contested.

### Research on free jazz improvisers' shared understanding

What is known about performers' shared understanding in free jazz improvisation? The variability of performers' characterizations of their own improvisational process in Pras' (2015a,b) qualitative interview analyses suggests that performers' understandings may differ substantially. In those interviews, some performers claimed avoiding thinking when they are improvising, while others described intense and controlled thought processes. At least in this set of interviews, the specific cultural backgrounds of the performers (all in the highly varied New York scene) did not seem to predict performers' self-reports, nor did any of the performers report that such differences would prevent them from improvising with other musicians who think differently. What was shared seemed to be more abstract. All mentioned some version of “being connected” while performing, which they expressed in various ways: as being connected with their body, mind, or instrument; as being connected with other musicians, the acoustics or the audience; and most broadly with a sense of transcendence, connecting to “the zone” or the universe. All interviewees also reported that they develop multiple musical languages and vocabularies to communicate with other improvisers.

Studies that focus more specifically on free jazz improvisers' shared understanding have demonstrated that improvisers' retrospective accounts of an improvisation can both converge and diverge from each other (Wilson and MacDonald, 2017). One quantified estimate of the potential degree of convergence is seen in Canonne and Garnier's studies of first-time trios and quartets of free jazz improvisers, who were asked to provide judgments of when they had “significantly changed their own musical production,” either concurrently during the improvisation using a foot pedal (Canonne and Garnier, 2015) or immediately afterwards (Canonne and Garnier, 2012). In both cases, performers' segmentation judgments (which moments they picked as representing a significant change) overlapped with at least one other performer's judgment about two thirds of the time. Overlap among all three or four musicians in the trios or quartets was substantially lower, at 37 and 12%. These findings suggest that there can be at least partial agreement across performers, but that it is far from complete.

Using a different method, Aucouturier and Canonne (2017) asked pairs of experienced music improvisers to musically communicate to one another five types of non-musical social intentions: being domineering, insolent, disdainful, conciliatory, or caring. At least when constrained to choosing among these five social intentions, the performers—along with other musically-trained and non-musically-trained listeners—were able to accurately recognize these relational intentions encoded in music, suggesting the potential for substantial shared understanding of at least certain aspects of music-making.

Using yet a different method, Canonne and Aucouturier (2016) asked 19 free improvisation students from the Paris Conservatoire to listen to 25 short improvised sound sequences (produced by other improvisers from the same community) and to judge, using a card-sorting procedure, the sequences' similarity. The finding was that improvisers who classified the sound clips more similarly were more likely to have had experience playing with each other, at least more than chance would predict. This suggests that thinking more similarly about improvised music correlates at least moderately with playing together more, although the direction of causality is of course unclear. The characterization of the finding as “play together, think alike” leads to the hypothesis that those who perform together should think more similarly to each other than those who do not.

### Research objectives and questions

In the current case study, we explore shared understanding among professional free jazz improvisers of international renown, and the extent to which that shared understanding is privileged relative to listeners with similar professional experience. To do this, we examine the extent to which two touring free jazz improvisers playing together for the first time agree with each other's characterizations of music they themselves have just played, and how their agreement with each other compares with their agreement with comments by listeners from the same performing community. We asked musicians to perform naturally, in a performance venue situation where they could fully see each other (unlike the improvisers in Schober and Spiro, 2014) and asked for their immediate retrospective accounts based on listening to their recorded performance. The idea was to take as a starting point active professional performers' experience and their characterization of that experience (Schiavio and Høffding, 2015; see also Pras and Lavergne, 2015; Wilson and MacDonald, 2016), leading to a rich set of moment-by-moment descriptions that can only apply to these performances, and the possibility of quantifying subsequent agreement with and about those statements.

Rather than using evaluative jury ratings that could characterize any performance (e.g., Thompson and Williamon, 2003; Wesolowski, 2016), we elicited statements of (at least one version of) what the performers and listeners themselves thought about the improvisation. Our approach is thus different from that in studies exploring performers' sense of identity as musicians (e.g., Wilson and MacDonald, 2012), performers' reflections on themselves as improvisers (e.g., Biasutti and Frezza, 2009), or of judgments of performers' appropriateness (e.g., Platz and Kopiez, 2013). It also differs from the approaches in studies from a range of disciplines that have focused on analyses of interaction and the musical structure in improvisation, with the goal of inferring what kinds of background knowledge and cognitive processes must be shared by performers in order to improvise together successfully (e.g., Monson, 1996; Healey et al., 2005; Gratier, 2008; Loehr et al., 2013; Keller, 2014, among many others).

As we see it, the range of possible outcomes is large. On the one hand, the unscriptedness of free jazz improvisation could lead performers to agree with each other less than improvisers on jazz standards do, because the absence of referent could make the material less straightforwardly predictable and because each performer works so hard on individuality that common understanding may be less likely. On the other hand, the fact that free jazz improvisation is so free-form could require performers to be *more* attuned to each other than performers in other genres, which could lead them to agree with each other's characterizations more. As Canonne and Garnier (2015) put it, developing shared understanding during the course of a collective free improvisation “is likely one of the regulative objectives of the musicians, as they have no referent to rely on.”

We also focus on what kinds of information free jazz improvisers choose to talk about, and which kinds of statements performers are more likely to agree upon. Common wisdom among free jazz improvisers—for some, central to their agenda—is that in free jazz improvisation there is no right or wrong, and that judgments of musical quality or success go against the importance of being able to let the music unfold without resistance (Werner, 1996). Based on this one might predict that free jazz improvisers should be less likely to produce or agree with evaluative statements than value-neutral statements. Another prediction, related to the longstanding distinction between the musical/esthetic/sounding outcome of an improvisation and the processes/interactions that led to it (see Arthurs, 2015b), is that free improvisers might be particularly leery of producing or agreeing with statements about performers' intentions—what they were thinking and what they meant while performing. They might instead be more likely to produce and agree with statements that characterize the music itself than statements that make claims about performers' thoughts and feelings.

The study thus asks the following research questions:

1. To what extent do free jazz improvisers comment on the same moments in the music they just performed, and to what extent does the content of their statements overlap?
2.
  - To what extent do free jazz improvisers endorse their performing partner's statements any more or less than their own?
  - To what extent do free jazz improvisers endorse each other's statements any more than statements made by commenting listeners from the same performance community?
3. To what extent are free jazz improvisers' *patterns of statement ratings* any more similar to their performing partners' patterns of ratings compared with commenting listeners' patterns of ratings?
4. Which kinds of statements are free jazz improvisers particularly likely to endorse and agree with each other about? In particular,
  - Do they endorse or have more similar patterns of ratings with statements about the music itself than with statements about the performers' intentions and thoughts?
  - Do they endorse or have more similar patterns of ratings with evaluatively-neutral statements than with evaluative (positive or negative) statements?

## Methods

The method involved a few straightforward steps. First, a saxophonist and a pianist freely improvised for about 40 min while being audio recorded. Immediately afterwards, both performers were interviewed independently about two recorded excerpts of their improvisation, with the purpose of eliciting concrete statements about particular moments in the recordings that could later be extracted for use in quantitative ratings. Two commenting listeners from the same performance community were also independently interviewed while listening to the same recorded excerpts, with the same purpose. Several months later, after listening to the excerpts again the performers and the commenting listeners independently rated their agreement, in an online survey, with 302 anonymized statements extracted from the interviews, using a 5-point scale from “strongly disagree” to “strongly agree.” Finally, the performers were independently asked to elaborate about why they had disagreed with any statements the other performer had agreed with.

Informed consent for participating and for releasing summary results was obtained from all participants, and consent for releasing audio recordings of the performances (available in Supplementary Materials) was obtained from the performers, following review of the procedure by The New School's Institutional Review Board (#1015-2014).

### Recruitment

Performers were recruited with the explicit aim of including successful professional touring artists who were part of the New York scene but from different cultural and geographical backgrounds and who had never played together. We recruited improvisers who were articulate and willing to share their experiences and to try something unconventional.

The performers were invited by email and informed that, if they agreed, they would improvise for about an hour and a half, that the improvisation would be recorded and that they would be interviewed separately about the performance. They were not given the prompts or the aim of the project. They were also told that they would later be asked to rate agreement with statements and listen to excerpts of the recording, and that their identities would remain anonymous throughout if they so chose.

The commenting listeners were recruited with the aim of including two additional successful professional touring artists from the same New York scene who would be articulate and willing to comment with specific detail about recordings. Because they would not be informed who the performers were, there was no constraint on whether they could have played with these performers before. The commenting listeners were invited by email and simply informed that they would be interviewed about recordings of other musicians; they were invited to participate in subsequent ratings during the consent process at the interview.

### Participants

The performers are renowned improvisers, pianist Matthew Shipp^1^ and a saxophonist who prefers not to reveal his or her identity, both based in New York City, of similar ages but from different cultural and geographical origins. They knew of each other's work but had never played together before.

The commenting listeners are also renowned improvisers based in New York City, drummer Todd Capp^2^ and another saxophonist who prefers not to reveal his or her identity, of different ages, cultural and geographical origins relative to each other and to the performers. We did not tell them who the performers were.

The performers received US $100 each for the recorded performance and the interviews to compensate for their time and involvement in the study, and an additional $100 each for their questionnaire ratings and subsequent elaboration about their disagreements. The commenting listeners received $50 each for their interviews and an additional $100 for their questionnaire ratings.

### Performance and interviews

#### Recording and excerpt selection

The two performers were recorded in November 2014 while improvising freely together without a pre-established plan for about 40 min in the Jazz performance space in Arnhold Hall at The New School. The second author and two interviewers constituted the audience, and the first author recorded the performance from the technical booth. Before the performers arrived, she set up an ORTF stereo system with Neumann KM84 for the piano and an AKG414 XLS in cardio for the saxophone, as well as an AKG414 XLS in omni to record the blend of the two instruments in the room. After a quick sound check to optimize the representation of both instruments' timbres in the recording and to balance the input levels of every microphone, the four tracks were mixed without equalization or dynamic compression and without changing the microphone gains and track levels throughout the performance. Therefore, individual dynamics of each performer were free of mixing manipulation and the set balance remained the same for every moment of the performance.

The first author quickly exported two excerpts from the performance, based on constraints that had been decided on beforehand, and with the goal that there would be about 10 min of music that the performers could discuss afterwards. The first segment was to begin with the opening—the first moments that these two performers would be playing with each other—and to continue for about 5 min. The second segment was to come from later in the performance and was to contrast with the first segment in some way—sonically, in terms of which performer seemed to be taking the initiative, or in some other way. Based on these constraints, Excerpt 1 went from the opening to 4:25; the ending was artificially created using a fade out, because there was no clear breaking point in the music. Excerpt 2, which started at 28:31 and lasted 6:19, was chosen both because it was preceded and followed by a few seconds of silence and because the saxophonist began this section solo, in contrast with Excerpt 1, which the pianist seemed to lead.

The excerpts were exported in wav format (CD audio quality) for use during the interviews. MP3 format versions of the two excerpts are available in the Supplementary Materials.

#### Performers' interviews

Immediately after the improvisation each performer was interviewed separately about the just-completed improvisation first from memory and then while listening to the two excerpts of the recording. The purpose of the interviews was not to create a corpus for qualitative analysis, but to prompt specific and detailed commentary and to elicit a set of ratable concrete statements that characterized the performance both in general and also in specific moments. This interview method (also used by Schober and Spiro, 2014) is similar in approach to Theureau's (2003, 2010) “self-confrontation” interview and “course-of-action” analysis methods from cognitive anthropology; see also Norgaard's (2011) exploration of thought processes of artist-level jazz musicians in which participants responded to prompts about sound recordings and transcriptions of the music that they had just improvised (Norgaard, 2011). It is also consistent with Sloboda's (1985) early call for immediate retrospective interviews using recordings and playback “with as many pauses and backtracks as required” to obtain “a detailed record of the conscious decisions involved in constructing the improvisation” (p. 149–150).

The interviewers were two graduate students from the classical music performance program (Mannes School of Music) at the College of Performing Arts at The New School. The pianist was interviewed by a classical violinist and conductor, and the saxophonist was interviewed by a classical pianist. We hoped that the interviewers' strong musical background in a different genre would make them effective in understanding the music vocabulary and references that the performers might use, as well as make them credible interlocutors for professional musicians. We also hoped that the interviewers' not belonging to the same musical community as the performers would lead to more explicit elaborations of tacit understandings that might not be considered necessary to mention within the community.

We trained both interviewers together on the interviewing techniques we wanted them to use in order to elicit detailed commentary about particular musical moments, with particular focus on being non-directive but also willing to probe further to get more detailed information. The interviewers' task was to elicit extractable statements that could later be rated, starting with the prompts in Table 1. They could let the performers start and stop the recording as they desired, listening multiple times if they liked, and interviewers were to elicit as much detailed commentary as possible; for example, if a performer smirked while listening to the recording but then offered no corresponding commentary, interviewers were to prompt for an explanation of the smirk. We encouraged the interviewers to get performers' explicit agreement about the time period that each description referred to (where in the recording).

**Table 1:** Performers' interview prompts.

General discussion prompts from memory	How would you describe the performance you two just gave?
	How easy or hard was it to play with your partner? Why (Please be as specific as you can)? Did this change over time?
	What did your partner do that struck you as particularly interesting or notable? (Please be as specific as you can, and about when during your playing)
Prompts during listening to excerpts	What did you think or feel during the performance?
	What do you think worked or what didn't?
	What did your partner do that struck you as particularly interesting or notable?
	Can you point at particular musical choices you made or your partner picked up on? That your partner didn't pick up on?
Ending questions	Is there anything you want to add about playing with the other—Anything particularly interesting or notable or enjoyable that you haven't already said?
	How well did you know your partner before today?

Beyond these instructions, we also provided the interviewers with some basic introductory information about free jazz improvisation and asked them to try to get beyond standard non-specific responses that musicians in this community tend to give to journalists about free jazz improvisation practices, e.g., *there's no such thing as right or wrong in free jazz, improvisers may react to any sound in the environment including non-musical sounds, they see themselves as able to cope with any situation*, or *they are not necessarily interested in what is beautiful or successful*. These interviews lasted for 43 min and 1 h 36 min.

#### Listeners' interviews

In February and March 2015, two commenting listeners were interviewed about the same two recorded excerpts by two other graduate students with musical backgrounds comparable to those of the other interviewers, a French horn player who interviewed the saxophonist, and a saxophonist who interviewed the drummer. The interviewers, who were trained separately for scheduling reasons, were trained using the same materials as the performers' interviewers, with slightly modified prompts that fit listening to the recordings (see Table 2). The interviews lasted for 1 h 6 min and for 1 h 23 min.

**Table 2:** Commenting listeners' interview prompts.

What do you think worked or what didn't?
What did either performer do that struck you as particularly interesting or notable?
Can you point at particular musical choices that one performer made that their partner picked up on? That their partner didn't pick up on?
What do you think the performers were thinking or feeling during the performance?

### Online survey

#### Statement selection

The four interviews were transcribed by graduate students at The New School for Social Research and an undergraduate at Parsons School of Design. From the transcripts, the authors extracted all statements that were specifically and unambiguously about the two recorded excerpts of this performance, excluding any statements about any other parts of the performance and any additional general statements about music, politics or the ideology of free jazz improvisation, as well as any identifying statements about the performers or facts that a listener could not know about with only the recording to go on (e.g., that the performers had not played together before, which was mentioned during the performers' interviews). We also excluded any statements that would require substantial rewording to be intelligible to an outsider or where we could not understand what the statement meant (e.g., *then it becomes like a dog biting his own tail*).

Of the 319 remaining statements, we excluded 5 which on further consideration would be too ambiguous to rate (e.g., the time period the statement referred to was ambiguous). We excluded 7 statements we judged as identical to other statements, retaining what we judged to be the clearer formulation. Finally, we excluded 5 statements that we judged as potentially insulting enough in this community to risk alienating the performer participants (e.g., statements of the type “*player X did not have ideas independent of player Y's*”). Even though the performers' reactions to these 5 statements might have been interesting, the remaining corpus of statements included enough negative evaluative material that their removal was unlikely to substantially change the pattern of findings.

This left a total of 302 statements by the interviewees available to be rated by the participants, with 185 statements that specifically referred to particular moments in the excerpts. To prepare the statements for rating without revealing the identities of their authors to the participants, we anonymized and de-gendered them, and we standardized the verb tenses, which varied even within interviews, with the convention that statements about specific moments in the music would be in present tense and general and global characterizations about the performance would be in the past tense. We removed disfluencies and repetitions, as well as ambiguous words like “really” that might or might not have been intended as intensifiers, and that, in our judgment, might confuse the ratable content (for example, if a rater agreed with the general idea but disagreed that it was extreme). In order to make definitive pronouncements ratable, we added “it sounds as if” to the start of 55 statements, so that raters would not need to judge definitively matters that they could not have access to. Similarly, in order to make judgments of liking or disliking ratable (as well as to depersonalize any negative evaluations and avoid offense), we transformed 3 statements that interviewees *liked* or *loved* or *didn't like* what had happened into statements that the moment *worked well* or *didn't work well*.

We also added time stamps into any statements that were about specific moments or time spans in the excerpts, based on the interviewees' own spontaneous descriptions of time spans or of specific related musical content, or on interviewees' responses to probing to clarify the time span any comment referred to.

Using these principles, the general statement about Excerpt 1 “we …were trying to really feel out each other's vocabulary” was transformed into *It sounds as if the performers were trying to feel out each other's vocabulary*. The statement about a specific moment in Excerpt 1 “This beginning I didn't like that much” was transformed into *Between 0:14 and 0:33, this beginning does not work well*. In this case, the time span was added based on the interviewee's immediately subsequent description of the musical content that they were referring to (“harmonic minor”).

Table 3 tallies the kinds of statements made by the different participants. Three of the interviewees gave every kind of statement, while Listener 2 (drummer listener) did not provide characterizations of specific moments in either excerpt. The Pianist and Listener 1 (sax listener) provided more usable comments, based on our criteria, than the saxophonist and Listener 2. Supplementary Table 1 provides the full list of 302 statements.

**Table 3:** Distribution of statements by each participant.

	**Saxophonist**	**Pianist**	**Commenting Listener 1**	**Commenting Listener 2**	**Total**
Characterizations of moments in Excerpt 1	18	16	34	0	68
Characterizations of moments in Excerpt 2	16	55	46	0	117
Overall characterizations of Excerpt 1	8	3	16	9	36
Overall characterizations of Excerpt 2	6	9	22	6	43
General comments about the performers	11	27	2	0	40
Characterizations of moments in both excerpts	34	71	80	0	185
Overall characterizations of both excerpts	14	12	38	15	79
Total	59	110	120	15	304

*Two characterizations were produced by two different interviewees (one general comment about Excerpt 1 and one general comment about Excerpt 2), and so the table includes 304 statements rather than 302*.

#### Survey implementation

A questionnaire consisting of six main sections was implemented in the Qualtrics platform for presentation in a web browser (Qualtrics, 2015), allowing participants to answer on their own devices at a time and place convenient for them and to take breaks if they liked. Participants were instructed that participating could take a couple of hours, and that they should be using a device and in a place where they could listen attentively without interruption before proceeding. They were also instructed that they could take breaks while answering if they needed.

After this instruction and consent screen, the questionnaire started with a screen requiring participants to listen to the first excerpt in its entirety before proceeding to answering questions about it; the survey was programmed not to allow the participant to continue to the next screen until the amount of time that it took for this excerpt to be played had elapsed. The survey then continued with the following sections:

- General characterizations of Excerpt 1, including 15 statements about the creative process and musical result, 8 statements about the pianist's performance, and 12 statements about the saxophonist's performance.
- Sixty-eight characterizations of particular moments in Excerpt 1, on three different screens.
- Fourteen general characterizations of how Excerpt 2 compared with Excerpt 1.
- General characterizations of Excerpt 2, including 14 statements about the creative process and musical result, 4 statements about the pianist's performance, and 10 statements about the saxophonist's performance.
- One hundred and seventeen characterizations of particular moments in Excerpt 2, on four different screens.
- Forty-one general statements about the performers^3^.

**Figure 1:**
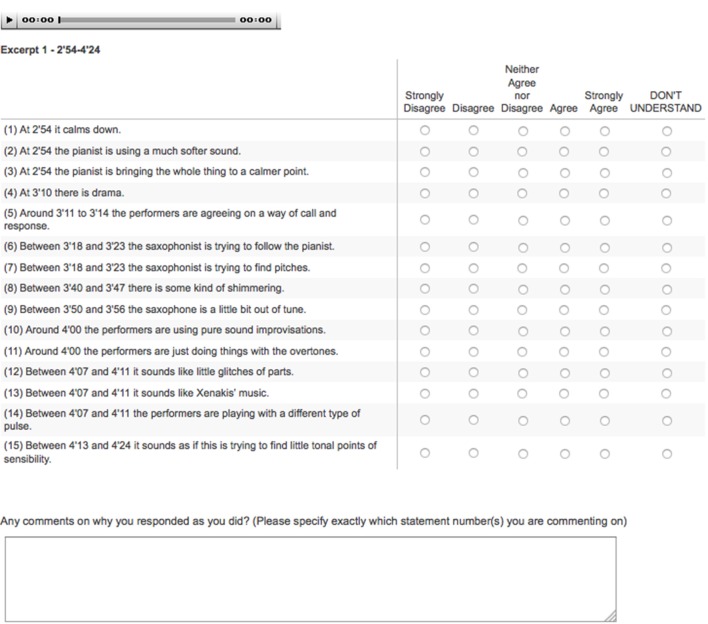
Example survey screen with statements and rating options.

In each section and on each screen, participants had the option of listening to the relevant audio excerpt again, and they were instructed to “feel free to listen again if you need to.” On each screen, participants were instructed to rate the extent to which they agreed or disagreed with the statements (on a 5-point scale, with labels “strongly disagree,” “disagree,” “neither agree nor disagree,” “agree,” and “strongly agree,” with the additional option of selecting “don't understand”). Each screen also included an open field in which participants could (optionally) add their own textual comments about why they responded as they had. See Figure 1 for an example screen, and the video available in Supplementary Materials for an illustration of particular survey questions corresponding with particular segments of the audio.

Before the first section about the second excerpt, an additional page required participants to listen to that excerpt in its entirety before proceeding.

The survey ended with a final section of 5 questions about the experience of responding to the survey: how long it had taken, how easy or hard it was, how enjoyable it was, whether the participant had taken breaks along the way, and if there was anything else the participant wanted to let the researchers know about their experience answering the questions.

### Retrospective rating and subsequent elaboration

In July 2015, all four participants completed the online survey, receiving $100 each for participating and agreeing to further explain any of their responses if needed. Three participants reported taking between 1 and 2 h to complete the survey with no break, and one commenting listener reported taking more than 2 h and having taken breaks. They ranged from reporting the experience to be “somewhat easy” and only “a little” enjoyable to “very hard” and “a lot” enjoyable.

From examining the ratings, we saw that there were 43 statements that one performer had disagreed with (rating “strongly disagree” or “disagree”) and that their partner had endorsed (rating “agree” or “strongly agree”). Following Schober and Spiro's (2014) method, we asked both performers to elaborate on their disagreement with these statements: 33 ratings for the saxophonist and 10 for the pianist. Both performers were invited to provide these elaborations via email, telephone, or in person as they preferred; both chose to respond via email, providing their elaborations in December 2015.

## Results

The participants gave substantive ratings for almost all statements; there were only 14 “don't understand” selections, between 2 and 6 per participant. The participants provided 66 optional written comments on their ratings, between 7 and 24 per participant. Almost all the comments elaborated or gave nuance to the ratings, and they demonstrated how seriously the participants took the task and how attentive they were to the wording of the statements. For example, one participant elaborated on an “agree” judgment with *Some of the pianist's playing was reminiscent of Mingus on piano* with “I first thought I disagreed but then listened to a few Mingus on piano excerpts and changed it to agree…there is something about that comment that rings true.” In only 3 cases (of 66 comments on 1,208 ratings) did a comment add a qualifier that could be argued to suggest the rating might not fully reflect the rater's opinion,^4^ and in only 2 cases did a comment show that a participant disagreed with a statement because they thought the time markers were off^5^.

### Statement overlap and similarity

**Figure 2:**
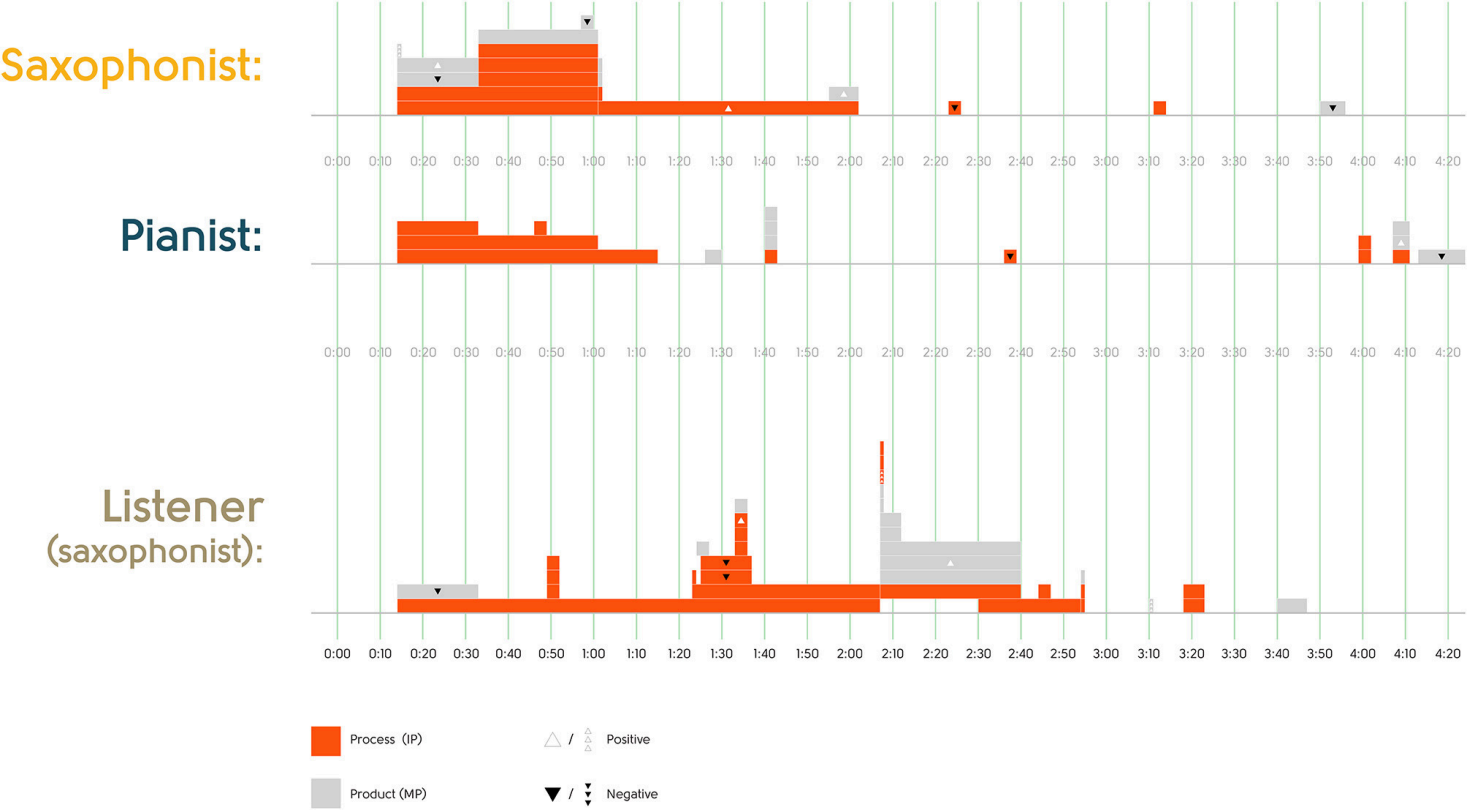
Timing of statements about specific moments in Excerpt 1.

**Figure 3:**
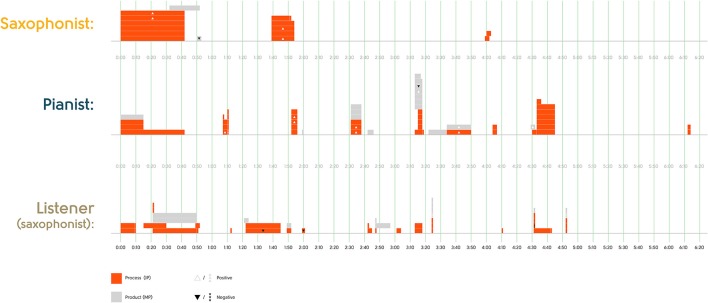
Timing of statements about specific moments in Excerpt 2.

The first part of Research Question 1 asks to what extent free jazz improvisers comment on the same moments in the music they just performed. Figures 2 and 3 plot the time spans referred to in the statements about particular moments by the performers and Listener 1. (There were no statements about particular moments by Listener 2). As visualized in the figures, although there was some temporal overlap in the moments that the participants described (particularly the beginnings of each excerpt), there was also substantial variability, with a number of moments only described by one participant.

To what extent did the content of statements about the same moments overlap? Recall that we selected statements for inclusion in the questionnaire based on our judgment that they were potentially non-identical in content, so in one sense these figures already plot non-overlapping content. Nonetheless, among the statements plotted we do see two statements about the same moment by different participants that could be seen as overlapping in content: (1) *Between 0*′*32 and 0*′*52 the music is kind of static* and *Between 0*′*21 and 0*′*50 the music is quite static*; and (2) *At 4*′*31 the pianist goes into a jazz style* and *From 4*′*33 the performers start playing jazz*. But the majority of statements about the same moment by different participants differ in content. (Whether the participants saw those different characterizations as complementary or incompatible is addressed in Research Questions 2–4).

Was there content overlap at a more abstract level—for example, in the kinds of statements that were made about particular moments? One immediately apparent difference in statements was that many focused grammatically on performers' actions—their improvisational process—while others focused on the musical product, with performers not as grammatical subjects of the actions^6^. The remaining statements (*n* = 19) described performers' musical background or knowledge. We therefore classified all 302 statements in the corpus into these three categories:

- Improvisational Process (IP) statements (*n* = 184), which focus on performers as agents (e.g., *From 1*′*33 the saxophonist is using the same line and kind of expanding a little bit on the idea*; *It sounded as if the performers were both trying to build lines at different dynamics*)
- Musical Product (MP) statements (*n* = 101), in which the performers are not the main grammatical subjects (e.g., *The music in this excerpt had more energy than the first excerpt*; *Between 2*′*07 and 2*′*40 there is a shimmering kind of idea*)^7^
- Background Knowledge statements (*n* = 19), e.g., *The pianist has facility in different voices and languages*; *It sounds as if the performers' vocabularies overlapped*

Figures 2 and 3, which plot the subset of statements about particular moments, make clear that both kinds of statements were made by all participants, and that there is no obvious overlap across participants in which kinds of statements were made. The distribution of IP vs. MP statements may simply result from our interview prompts, which pressed on performers' thoughts and actions, rather than reflecting what the participants may have spontaneously thought—free jazz improvisers may well avoid attributing intention or agency to each other (Pras, 2015b). In any case, this coding allows us to examine whether participants are more likely to endorse or agree with each other on statements about the music itself than statements about the performers' thoughts and actions (Research Question 4).

Another abstract kind of content overlap would be if the different participants overlap in evaluative statements—positive or negative characterizations—even if the precise content is different. We asked drummer Jim Black, who regularly performs in the New York City free jazz improvisation community, to evaluate each of the 302 statements for whether it would likely be perceived as positive, negative or neutral by improvisers within this community. He rated most of the statements (*n* = 210) as neutral, consistent with the norm in free jazz improvisation (and in talking about it) that there should be no right or wrong, but he did judge a minority of them as positive (*n* = 62) or negative (*n* = 30). For example, he judged *There was a fair amount of preconceived stuff* as negative within this community, and *There was drama and a sort of storyline* as positive. These judgments are consistent with characterizations by free jazz improvisers in this community that free jazz improvisation should not give listeners the feeling that the musical material has been prepared, and that free jazz improvisation should sound like “an instantaneous composition” that shows formal coherence (Pras, 2015a).

Figures 2 and 3 show how rarely evaluative statements were made about particular moments, and that there was no evident pattern of agreement in evaluation across the different listeners. But this coding, again, allows us to examine (Research Question 4) whether participants are more likely to endorse or agree with each other about evaluative or neutral statements.

These analyses suggest that there is little temporal overlap in the moments that these free jazz improvisers commented on, and that what they commented about does not overlap much at all, even at the more abstract level of positive vs. negative evaluation or whether they discuss improvisation processes or musical products (Research Question 1).

### Performers' statement endorsement

**Figure 4:**
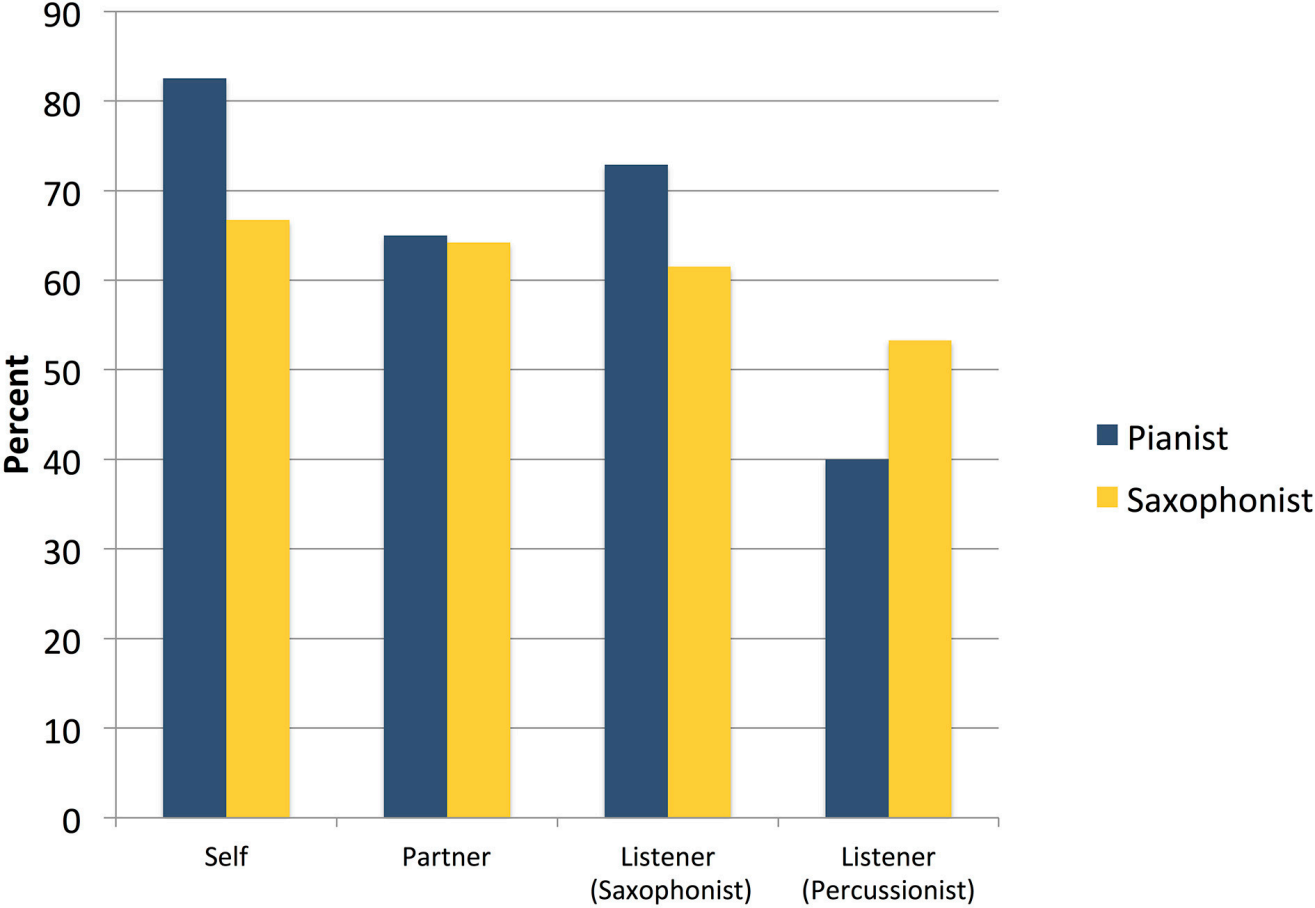
Performers' endorsement (percent with which they “agreed” or “strongly agreed”) of statements originally made by themselves, their partner, and the commenting listeners.

Research Question 2a asks whether free jazz improvisers endorse their performing partner's statements any more or less than their own, and Research Question 2b asks whether they endorse each other's statements any more than statements made by commenting listeners from the same performance community. As Figure 4 shows, both performers endorsed statements they themselves had generated more often than statements by their performing partner and either commenting listener, but with a different pattern. The pianist endorsed statements generated by the performing partner notably less than statements generated by one of the commenting listeners. The saxophonist endorsed statements by the performing partner and one of the listeners almost as much as their own statements, and with little difference between the listener and the performing partner. Both performers endorsed statements by the second listener least of all.

These patterns of endorsement both demonstrate, in different ways, that performers are not necessarily more likely to endorse statements by their performing partner than by a commenting listener. Consistent with the patterns of endorsement in Schober and Spiro's (2014) case study of improvisers on a jazz standard, these findings suggest that free jazz improvisers' interpretations are not necessarily privileged relative to the interpretations of a knowledgeable listener. But unlike in Schober and Spiro (2014), these musicians also endorsed some of their partner's statements more than another listener's.

### Performers' and listeners' interrater agreement

Research Question 3 asks whether free jazz improvisers' *patterns of statement ratings* are any more similar to their performing partners' patterns of ratings compared with commenting listeners' patterns of ratings. Table 4 presents interrater agreement between the performers and listeners as measured by the Cohen's Kappa statistic, where higher values indicate greater agreement, and is broken down by agreement about characterizations of moments in each excerpt, overall characterizations of each excerpt, and general comments about the performers. See Supplementary Table 1 for which particular statements garnered more and less agreement between the performers.

**Table 4:** Interrater agreement (Cohen's Kappa) between performers and listeners, using three rating categories: endorsement (4 or 5 on the 5-point scale), neutral (3), and dissent (1 or 2 on the 5-point scale).

	**Saxophonist Pianist agreement**	**Saxophonist Listener 1 agreement**	**Saxophonist Listener 2 agreement**	**Pianist Listener 1 agreement**	**Pianist Listener 2 agreement**
Characterizations of moments in Excerpt 1	**0.233**	**0.197**	**0.157**	0.036	**0.160**
	**(*****n*** = ***67*****)**	**(*****n*** = ***68*****)**	**(*****n*** = ***67*****)**	(*n* = *67*)	**(*****n*** = ***66*****)**
	**(*****p*** = ***0.010*****)**	**(*****p*** = ***0.028*****)**	**(*****p*** = ***0.066*****)**	(*p* = *0.695*)	**(*****p*** = ***0.051*****)**
Characterizations of moments in Excerpt 2	**0.277**	0.051	**0.264**	−0.021	0.075
	**(*****n*** = ***113*****)**	(*n* = *111*)	**(*****n*** = ***113*****)**	(*n* = *115*)	(*n* = *116*)
	**(*****p*** = ***0.000*****)**	(*p* = *0.463*)	**(*****p*** = ***0.000*****)**	(*p* = *0.758*)	(*p* = *0.137*)
Overall characterizations of Excerpt 1	0.192	−0.125	0.079	0.048	0.239
	(*n* = *15*)	(*n* = *15*)	(*n* = *14*)	(*n* = *15*)	(*n* = *14*)
	(*p* = *0.247*)	(*p* = *0.370*)	(*p* = *0.469*)	(*p* = *0.800*)	(*p* = *0.173*)
Overall characterizations of Excerpt 2	**0.627**	0.077	**0.413**	0.028	**0.377**
	**(*****n*** = ***11*****)**	(*n* = *12*)	**(*****n*** = ***11*****)**	(*n* = *13*)	**(*****n*** = ***12*****)**
	**(*****p*** = ***0.004*****)**	(*p* = *0.552*)	**(*****p*** = ***0.007*****)**	(*p* = *0.881*)	**(*****p*** = ***0.082*****)**
General comments about the performers	−0.176	0.073	**0.293**	−0.023	**0.346**
	(*n* = *40*)	(*n* = *40*)	**(*****n*** = ***40*****)**	(*n* = *40*)	**(*****n*** = ***40*****)**
	(*p* = *0.110*)	(*p* = *0.508*)	**(*****p*** = ***0.016*****)**	(*p* = *0.787*)	**(*****p*** = ***0.002*****)**
All statements	**0.271**	**0.088**	**0.233**	0.007	**0.134**
	**(*****n*** = ***294*****)**	**(*****n*** = ***294*****)**	**(*****n*** = ***293*****)**	(*n* = *298*)	**(*****n*** = ***296*****)**
	**(*****p*** = ***0.000*****)**	**(*****p*** = ***0.033*****)**	**(*****p*** = ***0.000*****)**	(*p* = *0.878*)	**(*****p*** = ***0.001*****)**

*Cells for which the kappa is statistically significant or marginal are highlighted in bold. The number of statements is slightly different across comparisons because we treated “don't understand” responses as missing data*.

For some kinds of statements—characterizations of the moments, and overall characterizations of Excerpt 2—performers' ratings agreed with each other more than chance^8^. The agreement on overall characterizations of Excerpt 2 was highest, at a level that in more traditional uses of kappa (interrater agreement among coders following training on the coding scheme) would be considered “substantial” (Landis and Koch, 1977), “fair to good” (Fleiss, 2003), or “moderate” (McHugh, 2012). Agreement on the characterizations of the moments was lower, at a level that can be considered “fair” (Landis and Koch, 1977), “poor” (Fleiss, 2003), or “minimal” (McHugh, 2012). For other kinds of statements—general statements about Excerpt 1 and general comments about the performers—performers' ratings did not agree with each other more than chance. Across the entire set of 302 statements, the overall kappa of +0.271 is not high, reflecting fair/poor/moderate agreement (depending on the interpretive scheme chosen) in traditional considerations of kappa.

How did performers' agreement with each other's ratings compare with their agreement with the two commenting listeners' ratings? As Table 4 shows, for each of the different kinds of statements and overall for all statements, agreement between at least one performer and one listener was quite close to the degree of saxophonist-pianist agreement. In fact, as measured by z-scores on the differences between performer kappas and performer-listener kappas, using Cohen's (1960) test, in 18 of 30 comparisons of performer kappas and performer-listener kappas there was no significant difference even with uncorrected alpha levels^9^. In some cases (general comments about the performers) there is *only* agreement between the performers and a listener, and no agreement with each other. Overall, the pattern is not consistent with a hypothesis that performers' understanding of each other is privileged relative to a non-performing listener, consistent with our prior evidence from improvisations on a jazz standard (Schober and Spiro, 2014, 2016).

### Endorsement of and interrater agreement with different kinds of statements

Research Question 4 asks whether free jazz improvisers are more likely to endorse or agree with each other's patterns of ratings on (a) statements about the music itself (MP statements) than statements about the performers' thoughts and actions (IP statements), and (b) value-neutral statements rather than evaluative (positive or negative) statements. Supplementary Table 1 details the categorization for each statement in the data set.

**Figure 5:**
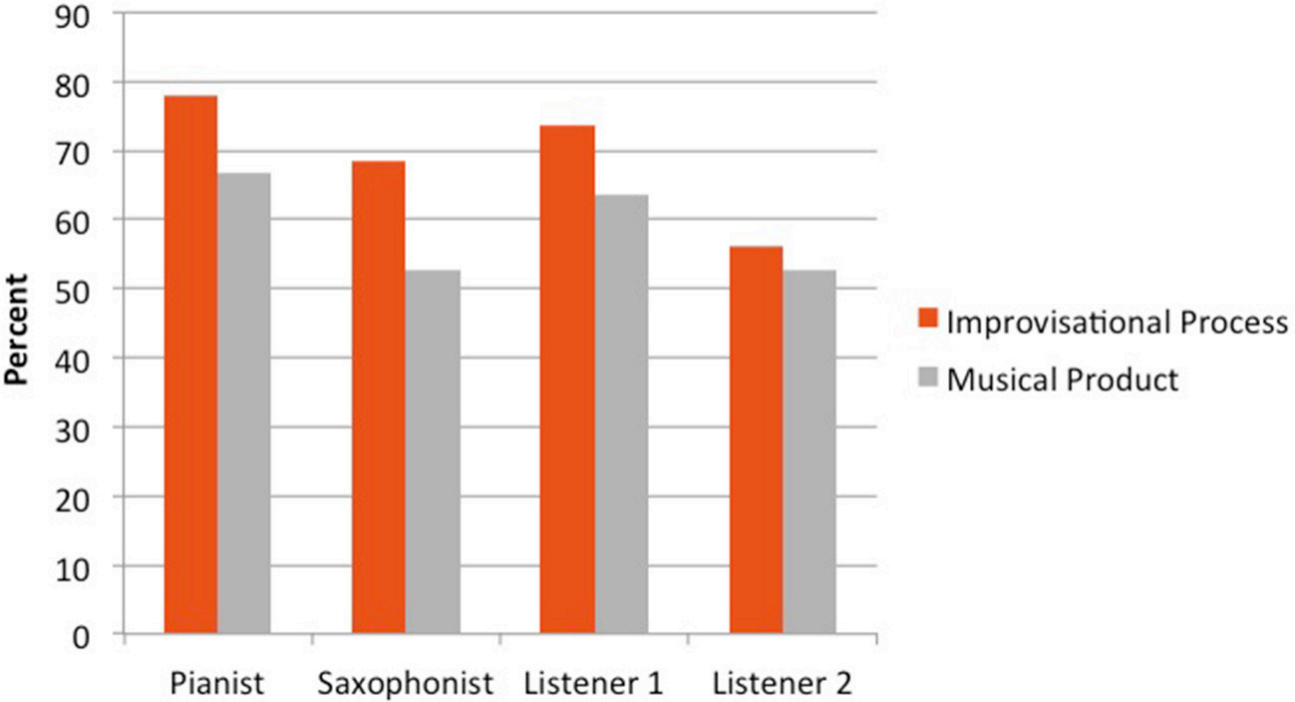
Performers' endorsement (percent with which they “agreed” or “strongly agreed”) of statements of different kinds: Improvisational Process statements (with performers explicitly marked as agents) and Musical Product statements (performers not marked as agents).

Figure 5 shows that all four participants endorsed IP statements more than MP statements. Table 5 shows that—seemingly conversely—as measured by Cohen's kappa the performers did *not* agree more about IP than MP statements; in both cases the level of interrater agreement is low, and the difference is not significant by Cohen's (1960) comparison test, *z* = −1.42. (The patterns of agreement between the performers and other listeners were different).

**Table 5:** Interrater agreement (Cohen's Kappa) between performers and listeners, using three rating categories: endorsement (4 or 5 on the 5-point scale), neutral (3), and dissent (1 or 2 on the 5-point scale).

	**Saxophonist Pianist agreement**	**Saxophonist Listener 1 agreement**	**Saxophonist Listener 2 agreement**	**Pianist Listener 1 agreement**	**Pianist Listener 2 agreement**
All statements	**0.271**	**0.088**	**0.233**	0.007	**0.134**
	**(*****n*** = ***294*****)**	**(*****n*** = ***294*****)**	**(*****n*** = ***293*****)**	(*n* = *298*)	**(*****n*** = ***296*****)**
	**(*****p*** = ***0.000*****)**	**(*****p*** = ***0.033*****)**	**(*****p*** = ***0.000*****)**	(*p* = *0.878*)	**(*****p*** = ***0.001*****)**
IP	**0.225**	**0.143**	**0.236**	0.007	**0.107**
	**(*****n*** = ***177*****)**	**(*****n*** = ***177*****)**	**(*****n*** = ***175*****)**	(*n* = *181*)	**(*****n*** = ***179*****)**
	**(*****p*** = ***0.000*****)**	**(*****p*** = ***0.007*****)**	**(*****p*** = ***0.000*****)**	(*p* = *0.902*)	**(*****p*** = ***0.031*****)**
MP	**0.371**	−0.004	**0.189**	−0.024	0.121
	**(*****n*** = ***98*****)**	(*n* = *98*)	**(*****n*** = ***99*****)**	(*n* = *98*)	(*n* = *98*)
	**(*****p*** = ***0.000*****)**	(*p* = *0.960*)	**(*****p*** = ***0.009*****)**	(*p* = *0.752*)	(*p* = *0.093*)
Positive statements	0.052	0.028	0.080	−0.097	0.113
	(*n* = *62*)	(*n* = *62*)	(*n* = *62*)	(*n* = *62*)	(*n* = *62*)
	(*p* = *0.570*)	(*p* = *0.767*)	(*p* = *0.392*)	(*p* = *0.271*)	(*p* = *0.250*)
Negative statements	**0.436**	−0.081	**0.445**	−0.171	**0.400**
	**(*****n*** = ***30*****)**	(*n* = *30*)	**(*****n*** = ***30*****)**	(*n* = *30*)	**(*****n*** = ***30*****)**
	**(*****p*** = ***0.001*****)**	(*p* = *0.467*)	**(*****p*** = ***0.000*****)**	(*p* = *0.145*)	**(*****p*** = ***0.002*****)**
Neutral statements	**0.238**	**0.093**	**0.206**	0.048	0.051
	**(*****n*** = ***202*****)**	**(*****n*** = ***202*****)**	**(*****n*** = ***201*****)**	(*n* = *206*)	(*n* = *204*)
	**(*****p*** = ***0.000*****)**	**(*****p*** = ***0.058*****)**	**(*****p*** = ***0.000*****)**	(*p* = *0.342*)	(*p* = *0.262*)

*Cells for which the kappa is statistically significant or marginal are highlighted in bold. The number of statements is slightly different across comparisons because we treated “don't understand” responses as missing data*.

What makes sense of this pattern is that for the performers, IP statements were more polarizing. They were more willing to endorse IP statements, but their judgments about them were equally likely to differ from their performing partner's than about MP statements. As we see it, this demonstrates that free jazz improvisers can be willing to endorse statements about thoughts and actions—perhaps contrary to what one might expect if free jazz improvisers refuse to see intentions as definitive or actions as having particular meanings. But their interpretations of those thoughts and actions are no more likely to be different from their performing partner's than their judgments about the musical content.

**Figure 6:**
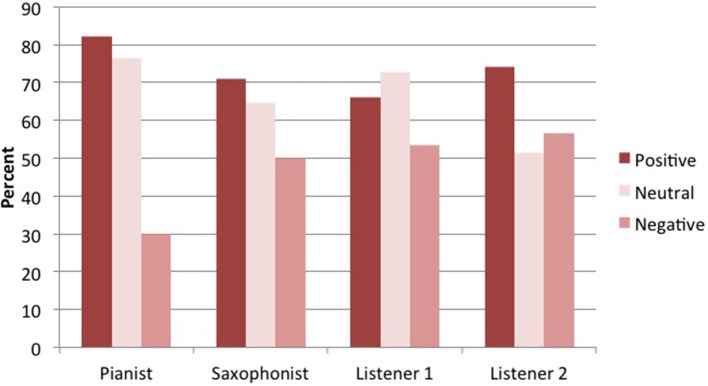
Performers' endorsement (percent with which they “agreed” or “strongly agreed”) of statements of different kinds: Evaluatively positive, neutral, and negative statements.

Research Question 4b asks whether participants are more likely to endorse or agree in their patterns of ratings about evaluatively neutral statements than positive or negative statements. Figure 6 shows that all participants endorsed evaluatively positive statements more than they endorsed negative statements, and that three of the participants endorsed evaluatively neutral statements at about the same level as positive statements. Table 5 shows that, despite this pattern of endorsement, they did not agree with each other about the positive statements at all. They were more likely to agree with each other about the negative statements^10^—most often (given the levels of endorsement) in rejecting the negative statements. This pattern is consistent with a free jazz ethos of rejecting the idea of there being anything “wrong” in free jazz, and little consensus on what is good; all our participants clearly demonstrate that way of thinking.

These findings show that form and content of characterizations of a free jazz improvisation can affect what improvisers endorse and agree with. In this case at least, they were more likely to endorse statements about performers' thoughts and actions than statements about the music itself, and they were more likely to endorse evaluatively positive than negative statements. But they did *not* agree with each other more about these kinds of statements—in the case of positive evaluation statements, they agreed less—suggesting that such statements can be more polarizing.

### Elaborations on disagreements

Table 6 lists the performers' emailed explanations for the reasons for their disagreement for the 43 statements on which they disagreed (33 for the saxophonist and 10 for the pianist). In only two cases did the performers now change their rating to agree; in two more the performers explained that they disagreed because they thought the timing was off (by 2 and 6 s) even if they agreed with the remaining content of the statement. This suggests that in most cases (39 of 43) the performers' disagreement with statements was reliable.

**Table 6:** Elaborations by performers on why they had dissented.

**Statement**	**About**	**Dissenter's elaboration**
**NOW AGREE**		
*The performers strongly showed that they were hearing what the other person was doing*.	Excerpt 1	I agree with it now.
*It sounds as if the performers were both very conscious of each other's space*.	Performers	I disagree with my disagreement.
**DISAGREEMENT ABOUT TIMING**		
*Around 1′25 there is kind of a cadence moment*.	Excerpt 1	Maybe at 1:22?? Otherwise I don't hear it.
*Right after 1′40 the performers go to the first section of flurries together*.	Excerpt 1	I hear flurries starting at 1:34.
**POSSIBLY NOT FULL DISAGREEMENT**		
*There was a certain sense of more sure-footedness after a minute*.	Excerpt 1	It seems somewhat true to me now.
*The performers reached a kind of peak and then it came down*.	Excerpt 1	I actually agree—I might have said disagree because there are a couple peaks we come off of as opposed to one explosion.
*In the very beginning the saxophonist was slightly behind*.	Excerpt 1	Not sure what “behind” means? tempo? dynamic? creativity? I think my intention was to play with a slower rate of development so as to balance the forward push of the piano.
*Around 0′47 the pianist is romanticizing things*.	Excerpt 1	I don't understand what that means, so I can't agree with it.
*The music in this excerpt had more energy than the first excerpt*.	Excerpt 2	I don't know what that means. That it demonstrates more energy? Or that more energy was expended by the performers? It's too vague for me to agree with.
*The music in this excerpt was more assertive than in the first excerpt*.	Excerpt 2	I don't think it's more “assertive”, whatever that means…
*Between 1′52 and 1′56 the saxophonist is phrasing*.	Excerpt 2	As opposed to what? I have no idea what that means, nor what it has to do with the interval I'm playing at that moment.
*Between 2′31 and 2′38 the pulse is “Stravinsky-esque.”*	Excerpt 2	I guess I can imagine hearing a little *Rite of Spring* in there, I just personally wouldn't describe it as Stravinsky-esque.
*Between 4′33 and 4′45 the performers are playing in a jazz pulse*.	Excerpt 2	What is jazz pulse? this is certainly not a typically “swinging” passage—and if that's not what is meant by “jazz pulse,” then I don't see how this could be more jazz than anything else.
*It sounds as if both performers tried to be maximalists and minimalists at the same time*.	Performers	I have no idea what that means.
*Some of the intervallic language of the saxophonist is based on Messiaen*.	Performers	I have not listened to Messiaen for years—other than the Quartet for the End of Time last thing I remember are clusters on an organ—hard for me to transfer that awareness to sax—but it's been so many years since hearing Messiaen—so it could be so.
*In this excerpt, the performers were spending more time exploring something, compared to the 1st excerpt*.	Excerpt 2	I do kind of agree—guess we sound more focused on a particular gesture if that's what you meant—think my disagree might have been more semantic.
*Between 0′00 and 0′15 after overblowing, the saxophonist goes to a minimalist style*.	Excerpt 2	I completely agree with this—that is what is being expressed.
*Between 0′00 and 0′42 the performers are spending more time exploring something (compared to the first excerpt)*.	Excerpt 2	I guess I agree—he starts with the Evan Parker inspired device and we distinctly explore that.
*Between 0′00 and 0′42 the pianist is coming in with a harmonic idea that has some distance from what the saxophonist is doing*.	Excerpt 2	I agree—for contrast—unison would be boring—but it's gesturally close enough to move the improv on.
*Around 0′50 the saxophonist is playing a bit more with the sound*.	Excerpt 2	I agree—seems like he is splitting the tone or trying to milk the tone—don't know if it's purposeful.
*Between 4′31 and 4′42 the pianist really goes into an idiom*.	Excerpt 2	I agree that there's a shift toward a certain idiom of voicing.
**REAL DISAGREEMENT**		
*The saxophone was very quiet and sort of polite*.	Excerpt 1	I disagree with the terms and the generalization —I think there is a lot of shifting between middle, fore and background going on.
*Between 0′14 and 1′01 the pianist is superimposing something over what the saxophonist is doing*.	Excerpt 1	I don't think it's an accurate or meaningful description. It seems the piano is providing the dominant more active idea and I'm playing a background with slower durations against it.
*At 1′01 there is sort of a silence*.	Excerpt 1	I don't hear a silence there—there's no attack but the piano is sustaining several sounds.
*Between 1′56 and 2′00 the dialog is interesting because the performers are not being locked into a certain harmonic theme*.	Excerpt 1	I think I disagreed because to me it is a continuation of the gesture before all be it there is no real harmonic underpinning at that point.
*Between 3′40 and 3′47 there is some kind of shimmering*.	Excerpt 1	I don't hear what I would call shimmering.
*At 1'07 it sounds as if the saxophonist is shifting to go into a certain space*.	Excerpt 2	I don't hear that. I think I'm leaving space for the piano's idea to come through.
*From 1′07 there is a little tension for the performers to find something together again*.	Excerpt 2	I think we're already in the thing we found and are just allowing it to stretch and breathe. I don't experience that as tension.
*At 1′12 the performers are back to shimmering*.	Excerpt 2	I don't hear any shimmering.
*Around 1′50 the pianist is using low octaves as they did in the first excerpt*.	Excerpt 2	I hear it as quite different from the use of low register in the first octave.
*1′51 is the end of the major 7 gesture on the saxophone*.	Excerpt 2	I hear that gesture informing everything I play until at least 2:45
*Between 1′52 and 1′56 the pianist is playing almost like a bass player*.	Excerpt 2	I don't hear that at all.
*At 1′59 the music gets into another pulse*.	Excerpt 2	I don't hear that in relation to anything happening in that moment. The way the pulse has been shifting around certain layers has been pretty consistent from the start and continues to be for a while.
*Between 2′31 and 2′38 the pianist opens up the harmonies vertically*.	Excerpt 2	I don't hear that.
*Between 2′31 and 2′38 the pianist opens up a group of harmonies that the saxophonist can shoot in and out of*.	Excerpt 2	He's repeating the same cluster and I'm repeating the same note, so I don't see how this statement could be an accurate description.
*Between 3′13 and 3′17 what the pianist is doing is funny*.	Excerpt 2	It didn't seem funny to me.
*Between 3′13 and 3′18 what the pianist did is funny*.	Excerpt 2	It still doesn't.
*Between 3′22 and 3′34 both performers' lines are independent*.	Excerpt 2	We're pretty independent in general. In this case, the motions mirror each other.
*It sounds as if both performers were skipping through ideas and shapes*.	Performers	I'm hoping we achieved something a little more coherent than “skipping” —but maybe I'm wrong. That's for someone else to judge.
*It sounds as if the saxophonist never loses sight of their instrument and what the instrument allows phrasing-wise as far as melodic possibilities*.	Performers	I think I'm often doing some very un-saxophonic things—I have other instruments in “sight”: viola, cello, flute, double reed, piano, percussion, etc.
*It sounds as if the performers come out of the same influences*.	Performers	I think it's safe to say any improv /jazz based musician would have a lot of the same influences—there is a certain pool of people to pull from—think we emphasize different things though.
*It sounds as if the performers are from different circles of musicians*.	Performers	Yes and no—we do come from different circles but I think enough common language exists to at least have the beginnings of a dialog.
*It sounds as if the performers' vocabularies overlapped but their processes are different*.	Performers	I don't hear that except in the vaguest way.

Of the remaining explanations, more than half either elaborate on the disagreement or simply restate it, suggesting that the disagreement is serious, e.g., the saxophonist explained their disagreement with *Between 0*′*14 and 1*′*01 the pianist is superimposing something over what the saxophonist is doing* with “I don't think it's an accurate or meaningful description. It seems the piano is providing the dominant more active idea and I'm playing a background with slower durations against it.”

The rest of the explanations suggest an openness to endorsing some version of the statement that is different from the one they first rated, but only if they interpret certain words differently than they originally did. For example, the saxophonist's explanation for disagreeing with *Between 4*′*33 and 4*′*45 the performers are playing in a jazz pulse* is “What is jazz pulse? This is certainly not a typically ‘swinging’ passage—and if that's not what is meant by ‘jazz pulse,’ then I don't see how this could be more jazz than anything else.” In a few cases, the performers now explained that they didn't understand the statement, even though they hadn't used the “don't understand” option on the rating scale in their initial rating. We interpret these explanations as polite responses despite continuing disagreement or as reflecting willingness to work at seeing the statements from another perspective, consistent with the accepting free jazz improvisation ethos. These explanations also may demonstrate that some of the initial ratings may be less reliable than they at first seem—that is, that when pressed the performer would be willing to bend in their judgment, to question their initial judgment, or to accept at least part of the interpretation that they nonetheless disagreed with overall.

Based on these explanations, we don't see a pattern in which of the disagreed-upon statements lent themselves to being open to reinterpretation, but the explanations do suggest that performers' interpretation can range in the degree to which they are malleable or resistant to change. We should note that this probably holds for the statements that the performers agreed upon as well: some statements might now be disagreed upon with additional listening to the recording and further contemplation.

Also worth noting is that this particular set of free jazz improvisers did not demonstrate the ideological basis of disagreements that we saw with the jazz standard improvisers in Schober and Spiro (2014). None of the explanations for disagreement here suggested that a performer was unwilling to endorse a particular kind of statement in principle, on the grounds that it violated a deeply held tenet about the nature of jazz; instead, these performers seemed particularly open to considering alternative perspectives—although not in every case. Some disagreements were fundamental.

## Discussion

The findings demonstrate that it is possible for free jazz improvisers to characterize their improvisation quite differently, selecting different moments to comment about and with little overlap in the content of their characterizations (Research Question 1). In this case study, these free jazz improvisers only sometimes endorsed statements by their performing partner more than those by a commenting listener from the same performance community, suggesting that their interpretations were not consistently privileged relative to the interpretations of a knowledgeable listener (Research Questions 2a and 2b). The performers' level of agreement with each other (patterns of endorsing or dissenting with statements across multiple ratings) was only moderate, and for a number of kinds of characterizations did not rise above chance levels. Performers' levels of agreement with each other were also not consistently higher than their agreement with commenting listeners from the same performance community (Research Question 3). Performers were more likely to endorse some kinds of characterizations than others: statements that grammatically focused on performers' thoughts and actions (more than statements focused on the music itself), and evaluatively positive (more than negative) statements. But these kinds of statements were polarizing; the performers did not agree with each other more on the kinds of statements they endorsed more. They were not more likely to agree with each other in their ratings of statements focused on the music itself (Research Question 4a), but they were more likely to agree about negative statements (Research Question 4b).

The pattern of findings is thus consistent with the pattern observed in improvisers on a jazz standard in Schober and Spiro (2014): less agreement among performing partners than might be expected, and little evidence that the performing partners agree with each other's judgments more than they do with those of knowledgeable listeners. Of course, this is a case study with one pair of performers and two commenting listeners; we don't know whether this pattern generalizes to all free jazz improvisers, to other knowledgeable listeners, or to listeners with less familiarity with the genre. Different recruitment or selection criteria for the performers and the commenting listeners (e.g., members of different performance communities or less experienced musicians) might lead to different patterns of results. Also, our method elicits one view of what performers and listeners think; we do not assume that our method elicits a full account of performers' cognition, and we recognize that each statement produced and rated in this study is created in particular situated ways.

But we find it striking that in a second case study in a freer genre of improvisation, and in different performance circumstances—performers knew who their partner was and how they play, and they could see them during the improvisation—we again see substantial discrepancy in performers' characterizations and judgments. How far this pattern extends is an open question. Additional studies using these or other materials with broader ranges of performers, performances, and listeners from different communities, along the lines of Schober and Spiro's (2016) audience study, will be an important next step.

Our interest in this study was assessing shared understanding by measuring the extent to which performers agree with each other's accounts of what they are doing, starting with artists' own characterizations—their individual rather than consensual interpretations. This led us to focus on particular characterizations of particular performances and moments in those performances, as opposed to general jury statements that could apply across many different performances. There are of course many other questions about the mental and social processes involved in improvisation practices that our method does not address. But we believe the method taps into independent thinking that wouldn't otherwise be visible for researchers, and that may not be visible to co-performers.

We see our method as allowing complementary insights into free jazz improvisers' shared understanding with work that focuses on, for example, judgments of segmentation (Canonne and Garnier, 2015) or characterization of the social relations embodied in the music (Aucouturier and Canonne, 2017). Our findings are consistent with those findings: at least this pair of improvisers shows *less than fully shared* understanding—agreement beyond chance, but not total agreement. The data do not suggest that these performers shared no understanding at all; it is hard to imagine how musicians could play together if they didn't share basic understandings about the nature of the activity, how to start and stop it, or which actions by the participants are licensed. But, just as free jazz improvisers do not necessarily agree on when a section of an improvisation they played starts or ends (Canonne and Garnier, 2015) or whether a particular recording clip demonstrated insolence or caring (Aucouturier and Canonne, 2017), they can disagree or even clash on many other characterizations of the music-making—particularly on statements about performers' thoughts and actions (more than statements about the music itself) and evaluatively positive (more than negative) statements.

Our findings provide further evidence on a *listeners-as-outsiders* hypothesis (Schober and Spiro, 2016) that listeners' different perspective on a performance (potentially leaving them out of some thoughts and feelings that the performers might share) may lead them to agree with other listeners' judgments more than with the performers'. (This hypothesis is independent of a *more-expert-listeners-understand-more-like-performers* hypothesis). In this case, our listeners—who were themselves highly expert performers from the same international community of performance, and so about as “insider” as an outsider can be—made some astute observations that demonstrate how insightful a listener-outsider can be, e.g., both listeners independently reported that the performers had never played together before (one said “It sounds as if these performers don't know each other very well” in the interview, and the other commented in responding to the survey that “They might know each other as friends but never have played together”). As outsiders, listeners may have insights on an improvisation that the performers themselves might not have, or even be able to have, perhaps akin to the kinds of insights that therapists might have on a marital interaction that a couple may be missing.

We see these results as adding to theorization about the nature of joint improvisation—and joint action more generally, given that most joint actions have an improvisatory quality in at least some sense. In Clark's (1996) formulation, joint actions can only succeed if both parties share sufficient common ground (mutual beliefs, knowledge, assumptions), which is based on their perceptual copresence, previous shared history, and community co-membership. Common ground needs to be shared “to a criterion sufficient for current purposes” (Clark and Wilkes-Gibbs, 1986; Clark and Schaefer, 1989; Clark, 1996), and this can vary in different arenas of joint action, with different participant goals, and different participant roles. In order to refer successfully in conversation, both parties need to share enough common ground to be able to pick out the same entity using the same words—but not necessarily more. We propose that in joint musical improvisation, similarly (even though coordinating on referring expressions isn't the goal), participants need a sufficient level of shared understanding to be able to carry out the joint activity at all, but how much agreement beyond that is necessary, possible, or even desirable is an open question (see Cross (2012) on the benefits for conflict-avoidance that music's indeterminacy allows). Based on our data, joint improvisation—at least of the quality found in these performances—can occur with substantial differences in interpretation of what just happened.

We propose that focusing only on what is common, rather than what is *not* shared, may be missing an important piece of the puzzle in understanding joint musical activity, not only in free jazz improvisation but also in improvisation on jazz standards (Schober and Spiro, 2014) and in performance of classical chamber music (Spiro and Schober, 2016). In the case of free jazz improvisation, as one reviewer of this paper put it, “Free improvisation presumably draws a lot of its power and meaning from the clash of conflicting musical ideas,” and at least some free jazz musicians “might consider a situation of complete agreement and harmony to be undesirable and counterproductive.” As we see it, whether musicians share understanding of what happened musically is logically independent of whether they see the music as embodying conflict or consensus; both parties might agree that what they just played embodied challenge and competitiveness, and they might disagree on characterizing what was harmonious. In any case, we see investigating musicians' points of disagreement as particularly likely to be informative and fruitful.

In some domains of joint action, partner predictability may lead to particularly satisfying or successful coordination, for example, in shaking hands, or in some kinds of partner dancing. But we speculate that—going beyond our data—interesting musical improvisation, or perhaps even interesting musical collaboration more generally, may actually *require* at least some difference in understanding between performers. If everything every party does is fully expectable and predictable, perhaps only predictable boring music will happen. Whether this is true of joint action more generally is less clear.

## Ethics statement

This study was carried out in accordance with the recommendations of The New School Human Research Protection Program with written informed consent from all subjects. All subjects gave written informed consent in accordance with the Belmont Report. The protocol was approved (determination #1015-2014) by the The New School Institutional Review Board.

## Author contributions

All three authors contributed substantially to the planning, analyses, and writing for this project. AP and MS collected the recordings and trained the interviewers.

### Conflict of interest statement

The authors declare that the research was conducted in the absence of any commercial or financial relationships that could be construed as a potential conflict of interest.

## Footnotes

^1^Matthew Shipp has been part of the New York downtown jazz scene since 1984. He started studying classical piano at the age of 5. His critically acclaimed discography, which highlights his work both as leader and sideman, as well as other biographical and performance details, can be found at http://www.matthewshipp.com.

^2^Todd Capp has been performing in the New York downtown/Brooklyn scene since the 1970's, and he currently performs with the Berlin-based trios *Oceans Roar 1000 Drums* and *Zug Zug*, and in New York with his own *Mystery Train*. His work is inspired by new perceptions of space and sound suggested by the embryonic AACM (Association for the Advancement of Creative Musicians) in Chicago in the 1960's. See http://www.toddcappmusic.com/ for more details.

^3^One additional statement from a prior study (“The pianist is open to doing whatever happens in the moment”) was included erroneously.

^4^The three qualified ratings were (1) a comment on a “strongly agree” rating with *The saxophonist has a very clear tone*: “Sometimes”; (2) a comment on an “agree” rating with two statements *(Between 0*′*33 and 1*′*01 the saxophonist is treating what the pianist is doing almost like a classical sonata where it becomes an accompaniment and the saxophonist is playing like a violin* and *Between 0*′*14 and 1*′*01 the saxophonist resists the pianist's invitation to be like a violinist playing a soaring melody over the accompaniment*): “The reality is closer to a compromise between [the two statements] - recognizing the invitation, resisting fixity of role”; (3) a comment on an “agree” rating with *It sounds as if the saxophonist was just following along*: “although I think the piano was leading for most of the time, it would seem a little unfair and negative to say the sax player was just following along - he seemed to be trying to make music and didn't have much choice than to follow along as there wasn't much response from the pianist.”

^5^The two disagreements because of timing were (1) a comment on *At 4*′*52 there is a cathartic moment from the saxophonist* of “catharsis happens closer to 5′10” and (2) a comment on *Between 1*′*39 and 1*′*51 the saxophonist is working with a major 7* of “Again timings are off: the Major 7 begins at 1′51 and goes to about 1′57.”

^6^Of course, these statements were elicited in interviews that have their own social and interactive dynamics, and so we cannot claim that this distinction in linguistic form reflects some pure essence of interviewees' stance or thinking, nor do we assume that musical processes and products can always be strictly distinguished, particularly in a genre that so prioritizes musical and interactive process (e.g., Bailey, 1993; Monson, 1996; Borgo, 2002). Nonetheless, the differences in articulation do reflect alternate perspectives on the phenomena under discussion by the participants themselves.

^7^This category includes 23 statements that might arguably be considered IP, in that they individuate an instrument or mention a player, e.g. *The saxophonist's intonation did not match the piano's tempered scale*; *Between 0:33 and 1:01 the pianist's playing is chromatic*. The pattern of findings reported here does not differ if we eliminate these statements from consideration or reclassify them as IP statements.

^8^Because our usage of kappa is unusual (kappa is most often used to measure interrater agreement using a coding scheme), we also simulated what chance agreement using kappa would look like for this many raters, statements, and categorizations of different kinds of statements (which varied in number), following a reviewer's recommendation for a previous case study (Patrick Healey). We did this by creating two data sets of 302 simulated ratings by four raters, where each data set included randomly generated integers between 1 and 3 with equal likelihood of occurring, to simulate our three levels of rating. We then repeated all the kappa comparisons reported in Table 4 on these data sets, with the idea that the highest value of these random kappas is a reasonable cutoff in order to set a high bar for assessing interrater agreement—for judging that any two parties agreed with each other more than chance would predict (see also Schober and Spiro, 2014, 2016). This precautionary analysis ended up showing the same pattern as the significance tests—that is, no significant kappas fell below the randomly generated levels.

^9^Thanks to a reviewer for suggesting these comparisons. The Cohen (1960) test calculates z-scores on pairs of kappas along with their standard errors to see if they differ significantly from each other. In our case, correction is needed for multiple comparisons (performers' kappas vs. all performer-listener combinations lead to 30 comparisons), although it is unclear what the most appropriate level of correction should be; correcting to an alpha level of 0.001 leaves only one significant difference, between kappas for performers and pianist-listener 1 on all statements. More importantly, the analysis treats pairs of raters as independent, which in our comparisons is certainly not an accurate assumption; but possible corrections for this (Williamson et al., 2000; Cao et al., 2016) would only decrease the likelihood of finding significant differences. In any case, the statistical evidence makes clear that most of the performer-performer kappas are not significantly higher than at least one of the listener-performer kappas.

^10^Using Cohen's (1960) test, *z* = −2.32 for positive vs. negative kappas. Again, this test assumes kappas are independent, which isn't the case here, and so we treat this finding with caution.
