# Supplementary material for: What About Their Performance Do Free Jazz Improvisers Agree Upon? A Case Study
Source: Front Psychol. 2017 Jun 26;8:966. doi: 10.3389/fpsyg.2017.00966 (PMC5483471; doi:10.3389/fpsyg.2017.00966)
Supplement: Supplementary file 1 [file Table1.pdf]

From Pras, A., Schober, M.F., & Spiro, N. (2017). What about their performance do free jazz improvisers agree upon? A case study. *Frontiers in Psychology, 8*:966. doi: 10.3389/fpsyg.2017.00966

Supplementary Table 1: The 302 statements and the performers' levels of agreement with them.
Kind of statement: Improvisational process (IP) statements focus grammatically on performers as agents; in musical product (MP) statements the performers are not the main grammatical subjects; background (BK) statements focus on performers' musical background or knowledge. Evaluative tone (positive, neutral and negative) was coded by drummer Jim Black, a member of this performance community.

Statement	Kind of statement	Evaluative tone
**Perfect agreement: Both performers "strongly agree" (N=6)**		
The pianist played thematic material that returned.	IP	Neutral
Between 0'00 and 0'42 this kind of merging between what the saxophonist is doing and the pianist is doing is cool.	IP	Positive
Between 4'33 and 4'45 it sounds as if the performers are mentally locked together.	IP	Neutral
It sounds as if the performers were both very conscious of trying to make music out of the experience.	IP	Positive
It sounds as if the performers were trying to find some point of intersection.	IP	Neutral
The pianist has facility in different voices and languages.	BK	Neutral
**Perfect agreement: Both performers "agree" (N=104)**		
There was drama and a sort of storyline.	MP	Positive
It sounds as if it was the first time the performers played together.	IP	Negative
The piano was dominating the sound in the mix.	MP	Negative
It sounds as if the pianist had certain ideas that they developed.	IP	Positive
It sounds as if the pianist pretty much stuck with those ideas.	IP	Neutral
The saxophonist was finding how to stay in tune with the piano.	IP	Negative
In the very beginning the saxophonist was trying to match what the pianist was doing.	IP	Neutral
At 0'14 the first sound together works very well.	MP	Positive
Between 0'14 and 0'33 there is a kind of willfulness to find a solution rather than just listening to the sound and the blend in the room.	MP	Positive
Between 0'14 and 0'33 the pianist is sustaining a romantic type of thing.	IP	Neutral
Around 0'50 the saxophonist is trying to match the pianist's chords.	IP	Neutral
Between 0'14 and 1'01 the saxophonist resists the pianist's invitation to be like a violinist playing a soaring melody over the accompaniment.	IP	Neutral
Between 0'14 and 1'01 the saxophonist is kind of floating.	IP	Neutral
At 1'01 a new movement starts.	MP	Neutral
At 1'23 the performers hit a note together.	IP	Neutral
From 1'33 the saxophonist is coming into their own.	IP	Positive
From 1'33 the saxophonist is using the same line and kind of expanding a little bit on the idea.	IP	Neutral
From 1'33 the saxophonist is generating a little bit more rather than just reacting.	IP	Neutral
Between 1'36 and 1'40 this is the saxophonist's first flurry.	MP	Neutral

Right after 1'40 the section of flurries breaks away from the romantic type of thing.	MP	Positive
Between 1'23 and 2'07 the saxophonist is trying to elaborate a little bit.	IP	Negative
At 2'07 the landscape changes.	MP	Negative
Right after 1'40 the section of flurries breaks away from the romantic type of thing.	MP	Neutral
Between 1'23 and 2'07 the saxophonist is trying to elaborate a little bit.	IP	Neutral
At 2'07 the landscape changes.	MP	Neutral
At 2'07 the saxophonist uses a different sound.	IP	Neutral
At 2'07 the pianist is reacting to what the saxophonist is doing.	IP	Neutral
At 2'07 the performers are not doing exactly the same thing but they are playing the same ideas.	IP	Positive
Between 2'07 and 2'12 there is a little bit more drama.	MP	Neutral
Between 2'07 and 2'12 there is a little bit of a peak.	MP	Neutral
Around 2'45 the pianist goes back to dark notes.	IP	Neutral
At 2'54 it calms down.	MP	Neutral
At 2'54 the pianist is using a much softer sound.	IP	Neutral
At 2'54 the pianist is bringing the whole thing to a calmer point.	IP	Neutral
At 3'10 there is drama.	MP	Positive
Around 3'11 to 3'14 the performers are agreeing on a way of call and response.	IP	Neutral
Between 4'07 and 4'11 it sounds like little glitches of parts.	MP	Neutral
Between 4'07 and 4'11 the performers are playing with a different type of pulse.	IP	Neutral
There was more flow than in the first excerpt.	MP	Positive
The music in this excerpt was a little more balanced between the two musicians than in the first excerpt.	MP	Positive
This excerpt sounded more self-assured than the first excerpt.	MP	Positive
This excerpt gave the feeling of a larger form.	MP	Neutral
There was no problem of intonation in this excerpt.	MP	Positive
The piece had a certain thematic thing.	MP	Positive
It sounded as if the performers were trying to switch a tone here and there in a minimalist style.	IP	Neutral
It sounded as if the pulse held both performers' lines together.	MP	Neutral
The performers moved quite together through peaks and valleys.	IP	Neutral
In this excerpt, the saxophonist was playing quite repetitive phrases.	IP	Neutral
Although the saxophonist was denser than in the opening excerpt, he also left space to form new ideas.	IP	Positive
The saxophonist picked up on a lot.	IP	Neutral
The saxophonist could have picked up on more of the pianist's subtleties and movements.	IP	Negative
Between 0'00 and 0'09 the saxophonist plays breath sounds.	IP	Neutral
At 0'21 the performers are going into a shimmering thing.	IP	Neutral

Between 0'21 and 0'50 the music has a minimalist vibe.	MP	Neutral
At 0'50 the performers change pitches.	IP	Neutral
From 1'07 it sounds as if the saxophonist finds a way to vary the little tonal thing that he is doing.	IP	Neutral
From 1'07 while the pianist is still thinking in terms of minimalism, the saxophonist starts phrasing.	IP	Neutral
At 1'10 the saxophonist starts phrasing.	IP	Neutral
At 1'10 it sounds as if the pianist is still thinking in little minimalist nuggets.	IP	Neutral
At 1'10 the saxophonist varies the tonal pattern.	IP	Neutral
At 1'10 the saxophonist creates a different timbre by playing on their keys.	IP	Neutral
At 1'10 when the saxophonist plays on their keys creating a whole different timbre, it indicates that there's a whole set of other possibilities being thrown out there.	IP	Positive
From 1'21 there is the same phrase a few times.	MP	Neutral
Around 1'50 the arpeggios suggest that the pianist has a classical background.	MP	Neutral
Between 1'22 and 1'45 the saxophonist repeats intervallic shapes.	IP	Neutral
Between 1'39 and 1'54 the saxophonist is alternating between a static space around E flat and the repetition of a phrase.	IP	Neutral
Between 1'39 and 1'54 the saxophonist is creating a little form.	IP	Neutral
Between 1'39 and 1'54 the saxophonist is repeating their ideas to support what the pianist is doing.	IP	Positive
Between 1'52 and 1'56 it sounds as if the saxophonist is really comfortable with the pulse.	IP	Positive
Between 1'52 and 1'56 it sounds as if the saxophonist is getting funky with the pulse.	IP	Positive
Between 2'31 and 2'38 the pianist's harmonic playing is like a continuum.	MP	Neutral
Between 2'31 and 2'38 the pulse is centered.	MP	Neutral
Between 2'31 and 2'38, the pianist is making their harmonies work with whatever the saxophonist plays.	IP	Positive
At 2'42 the saxophonist starts playing a little bit of a melody.	IP	Neutral
Between 2'42 and 2'46 this phrase is funny.	MP	Neutral
From 2'42 the saxophonist repeats the same melody several times.	IP	Neutral
At 2'47 the pianist picks up on the saxophonist's melody.	IP	Neutral
Between 2'47 and 2'57 there is a moment of calm.	MP	Neutral
At 2'47 the shimmering thing starts quietly and then grows.	MP	Neutral
Around 3'02 the pianist uses the left hand octave to create big drone-like lines.	IP	Neutral
At 3'18 the saxophonist's tone and the way they play the end of their phrase announces that the section is going to stop.	IP	Neutral
Between 3'13 and 3'18 the pianist is trying to make it melodic.	IP	Neutral
Between 3'13 and 3'18 what the pianist does sounds nice.	MP	Positive
At 3'24 there is a beautiful beat.	MP	Positive

At 3'24 the performers are playing melodic lines.	IP	Neutral
At 3'24 the music has a searching quality.	MP	Neutral
At 3'24 the performers are not imitating each other.	IP	Neutral
At 3'24 the music has a conversational aspect.	MP	Neutral
At 3'24 there is a certain counterpoint element.	MP	Neutral
At 3'24 the performers are sort of following each other.	IP	Neutral
Between 3'34 and 3'50 it sounds as if the performers are not trying to force anything.	IP	Positive
Between 3'34 and 3'50 it feels really natural.	MP	Positive
From 4'00 the performers start playing different approaches to D minor with different kinds of cadences to move harmonically to.	IP	Neutral
Around 4'05 the pianist is not trying to mirror the saxophonist.	IP	Neutral
Between 4'29 and 4'32 the music is very beautiful.	MP	Positive
At 4'52 the saxophonist is taking charge.	IP	Neutral
At 4'52 the saxophonist is using harmonics.	IP	Neutral
At 4'52 the saxophonist is digging more into extended techniques.	IP	Neutral
At 4'52 what the saxophonist is doing is a little more expressive.	MP	Positive
At 6'12 to 6'14 the pianist picks up on the saxophonist's last tone and incorporates that in their chord.	IP	Neutral
At 6'12 to 6'14 the pianist put an exclamation point on the ending.	IP	Neutral
It sounds as if the performers' sensibility allowed the music to shape itself.	MP	Neutral
The saxophonist's phrasing involves kind of minimalism.	MP	Neutral
The saxophonist's phrasing is jazz-oriented.	MP	Neutral
The saxophonist melodically phrases like Paul Desmond.	IP	Neutral
It sounds as if the performers are from different circles of musicians with some overlap.	BK	Neutral
It sounds as if the performers' backgrounds were basically jazz.	BK	Neutral
**Substantial agreement: One performer "strongly agrees" and the other "agrees" (N=45)**		
It took the performers a while to get used to the acoustics of the room and to get a good blend.	IP	Negative
In the very beginning the saxophonist was trying to find harmonically what was going on.	IP	Negative
It sounded as if the performers had a good time with each other.	IP	Positive
It sounded as if the pianist has some classical roots.	BK	Neutral
At 4'31 the pianist's playing has a touch of swing.	MP	Neutral
Between 4'33 and 4'45 it sounds as if the performers are aware of their phrases looping.	IP	Neutral
Between 4'33 and 4'45 the performers are both playing ahead of the beat.	IP	Neutral
Between 4'33 and 4'45 it sounds as if the performers have the exact same gesture.	IP	Neutral
Between 4'33 and 4'45 it sounds as if both players know where the pulse is.	IP	Neutral
It sounds as if the performers were both really conscious of not trying to force something to happen.	IP	Positive

It sounds as if the performers were both trying to really listen.	IP	Positive
The performers were not really playing off sounds.	IP	Neutral
The performers were playing off phrasing.	IP	Neutral
The performers were playing off rhythmic contours.	IP	Neutral
Most of the music had a pulse behind it rooted in the jazz sensibility.	MP	Neutral
The pianist has great ears.	BK	Positive
The pianist responds quickly.	IP	Positive
The pianist shifts harmony a lot.	IP	Neutral
It sounds as if the pianist is a very sensitive musician.	BK	Positive
It sounds as if the pianist was trying to be musical.	IP	Positive
The pianist approaches the instrument in a romantic way.	IP	Neutral
It sounds as if the performers were trying to feel out each other's vocabulary.	IP	Neutral
It sounds as if the performers were looking for a common shape.	IP	Neutral
It sounds as if the pianist was not going to be thrown off course or detoured by anything the saxophonist might have done.	IP	Neutral
If the saxophone were brought up a little more, that would equalize the roles.	MP	Positive
The saxophonist was still being very lyrical when using big intervals.	IP	Positive
At 1'01 the pianist breaks the pattern.	IP	Neutral
Between 0'14 and 1'15 it sounds as if the pianist is hyper-conscious about whether or not the saxophonist wants to play romantically.	IP	Neutral
From 1'33 it's a little bit more of a conversation.	MP	Neutral
Between 2'07 and 2'40 there is a shimmering kind of idea.	MP	Neutral
Between 2'07 and 2'40 there is a nice texture.	MP	Positive
Between 2'30 and 2'54 the pianist is playing low clusters.	IP	Neutral
This excerpt was more relaxed than the 1st one.	MP	Neutral
It sounded as if the saxophonist was comfortable throwing something out there and seeing where the pianist was going to go with it.	IP	Positive
It sounded as if the performers were trying to take a little motif and see if they hit on something sort of similar.	IP	Neutral
It sounded as if the performers were both trying to build lines at different dynamics.	IP	Neutral
Between 0'00 and 0'09 the saxophonist plays quiet multiphonics.	IP	Neutral
Between 0'00 and 0'42 what the pianist is doing is related to what the saxophonist is doing but is not immediately adapting to it.	IP	Neutral
Between 0'00 and 0'42 both performers are allowing the difference between their two ideas to just stay there.	IP	Neutral
Between 0'00 and 0'42 it sounds as if both performers are already trying to figure out little variations.	IP	Neutral
Between 0'00 and 0'42 the saxophonist is playing a repetitive looping phrase with alternate fingerings.	IP	Neutral

From 3'15 it sounds as if the saxophonist is trying to romanticize the note.	IP	Neutral
From 3'15 it sounds as if the saxophonist is getting some sound variations off of a very small segment.	IP	Neutral
From 3'15 it sounds as if the pianist is trying to fill out what the saxophonist is doing.	IP	Neutral
At 4'31 the pianist plays a 7 sharp 9 chord.	IP	Neutral
It sounds as if the performers are coming from a different place.	BK	Neutral
**Perfect agreement: Both performers "disagree" (N=22)**		
It sounds as if the performers adapted to what they thought the other person was doing with too much willingness.	IP	Negative
In the very beginning the pianist's chords moved quite quickly.	MP	Neutral
Some of the pianist's playing was reminiscent of Mingus on piano.	MP	Positive
Between 0'14 and 0'33 this beginning does not work well.	MP	Negative
Between 0'33 and 1'01 the pianist's playing is chromatic.	MP	Neutral
Between 0'14 and 2'07 the pianist is being incredibly busy.	IP	Neutral
Around 4'00 the performers are using pure sound improvisations.	IP	Neutral
Around 4'00 the performers are just doing things with the overtones.	IP	Neutral
There was a fair amount of preconceived stuff.	MP	Negative
The music in this excerpt had an AABA form.	MP	Neutral
It sounded as if the pianist is a contemporary straight-ahead jazz player.	BK	Neutral
The saxophonist's intervallic language was reminiscent of Steve Coleman.	MP	Neutral
Some of the saxophonist's intervallic language was based on Messiaen's scales.	MP	Neutral
Between 0'15 and 0'30 the performers are working on the same shimmering kind of thing as in the first excerpt.	IP	Neutral
Between 0'21 and 0'50 the music is quite static.	MP	Neutral
Between 1'59 and 2'01 the performers are resting on the shimmering patterns a little too much.	IP	Negative
Between 1'22 and 1'45 the saxophonist is playing using preconceived ideas.	IP	Negative
From 3'15 the performers get back to minimalism.	IP	Neutral
Between 3'13 and 3'18 what the pianist does makes it like very modal jazz.	MP	Neutral
Between 3'13 and 3'18 what the pianist does sounds contrived.	MP	Negative
Before 4'33 the performers are doing things that are based on overtones.	IP	Neutral
At 4'31 it sounds as if the pianist falls back into something they know.	IP	Negative
**Substantial agreement: One performer "strongly disagrees" and the other "disagrees" (N=7)**		
It sounds as if the saxophonist was just following along.	IP	Negative
All the way through this excerpt, the piano was in general very dense.	MP	Neutral

In this excerpt, it sounded as if the saxophonist included preconceived material.	IP	Negative
Between 0'00 and 0'15 the saxophonist starts off overblowing.	IP	Neutral
The beginning sounds as if the performers were playing over some fixed chords or scales.	IP	Neutral
Between 0'14 and 0'33 it is a little bit too soon to embark on something like this.	MP	Negative
After 0'50 after the performers let go of a static space, there was a moment where it was not really gelling.	MP	Negative
**Perfect agreement: Both performers "neutral" (N=7)**		
It sounds as if the pianist's approach was "here is what it is, find me."	IP	Negative
The saxophonist's intonation did not match the piano's tempered scale.	MP	Negative
It sounds as if the saxophonist had fun.	IP	Positive
Right after 1'40 the section is more linear.	MP	Neutral
Right after 1'40 the section is more jazz.	MP	Neutral
This excerpt was more interesting than the first excerpt.	MP	Positive
Between 3'13 and 3'18 it sounds almost like two little bells.	MP	Neutral
**Possible agreement: One performer "agrees" and the other "neutral" (N=34)**		
If the saxophone were brought up a little more, that would equalize the roles.	MP	Positive
Between 0'14 and 1'15 it sounds as if the pianist is hyper-conscious about whether or not the saxophonist wants to play romantically.	IP	Neutral
Between 2'07 and 2'40 there is a shimmering kind of idea.	MP	Neutral
Between 3'18 and 3'23 the saxophonist is trying to find pitches.	IP	Neutral
Between 4'07 and 4'11 it sounds like Xenakis' music.	MP	Positive
In this excerpt more elements come from the saxophonist compared to the other excerpt.	MP	Neutral
Between 1'39 and 1'54 the saxophonist is repeating their ideas to give them more consistency.	IP	Positive
Between 3'13 and 3'18 the saxophonist plays a line without trying to find the pianist's chords.	IP	Neutral
Around 4'00 it sounds as if the pianist heard D minor tonality.	IP	Neutral
Around 4'05 the pianist is playing off the push-pull of the saxophonist's phrasing.	IP	Neutral
At 4'42 the pianist abandons what they started at 4'31.	IP	Neutral
It sounds as if the saxophonist is a very sensitive musician.	BK	Positive
The saxophonist is working off of tonal points.	IP	Neutral
In the case of this saxophonist there is a nice sensibility of the beauty of the sound of the instrument.	MP	Positive
The saxophonist has a very clear tone.	IP	Neutral
It sounds as if the performers have crossed paths before.	BK	Neutral
It sounds as if the performers were trying to find each other sometimes more successfully than other times.	IP	Neutral
It sounds as if these performers don't know each other very well.	BK	Negative

It sounds as if these performers may have a different language.	BK	Neutral
The saxophone was warming up.	MP	Negative
At some moments the saxophone was a little out of tune.	MP	Negative
Around 0'50 the pianist is putting out a lot of different chords.	IP	Neutral
Between 0'33 and 1'01 the pianist is working through a motive pattern.	IP	Neutral
Between 0'33 and 1'01 the saxophonist is treating what the pianist is doing almost like a classical sonata where it becomes an accompaniment and the saxophonist is playing like a violin.	IP	Neutral
Between 3'18 and 3'23 the saxophonist is trying to follow the pianist.	IP	Neutral
Between 4'13 and 4'24 it sounds as if this is trying to find little tonal points of sensibility.	MP	Negative
It sounded as if the pianist was feeling more comfortable in this excerpt than in the first excerpt.	IP	Positive
This excerpt was more enjoyable than the first excerpt.	MP	Positive
At 0'21 the performers are going into a swirling thing.	IP	Neutral
Between 1'52 and 1'56 the saxophonist is not mirroring or playing off the pianist's line.	IP	Neutral
Between 3'13 and 3'18 the saxophonist starts playing melodies.	IP	Neutral
The tone of the saxophone is beautiful.	MP	Positive
It sounds as if the saxophonist is not the type of player who will just force themselves on top of the piano.	IP	Positive
It sounds as if the performers' vocabularies overlapped.	BK	Neutral
**Possible agreement: One performer "strongly agrees" and the other "neutral" (N=9)**		
Between 1'25 and 1'37 the saxophonist is trying to find what's going on.	IP	Negative
Between 1'25 and 1'37 the saxophonist is trying to find the pitches.	IP	Negative
Between 0'00 and 0'15 the saxophonist's playing at the start sounds as if it is influenced by Evan Parker.	MP	Neutral
At 4'31 the pianist goes into a jazz style.	IP	Neutral
From 4'33 the performers start playing jazz.	IP	Neutral
The performers were playing off melodic ideas that generated some counterpoint.	IP	Neutral
The saxophonist has facility in different voices and languages.	BK	Positive
Between 1'01 and 2'00 the performers are finding the form of this section together.	IP	Positive
The music in this excerpt had an ending.	MP	Neutral
**Possible agreement: One performer "disagrees" and the other "neutral" (N=10)**		
The performers moved very fast from one idea to the next idea.	IP	Neutral
In the very beginning it sounded very much piano-led.	MP	Neutral
Between 0'57 and 1'00 this note on the saxophone does not work well.	MP	Negative
Around 2'37 it sounds as if the performers are still trying to feel each other out.	IP	Negative
The music in this excerpt could be structured in 2 pieces.	MP	Neutral

Between 0'32 and 0'52 the music is kind of static.	MP	Neutral
At 2'07 it gets darker.	MP	Neutral
Around 2'24 it sounds as if the saxophonist is trying to find a way to hook up.	IP	Negative
It sounded as if the saxophonist had listened to a lot of Tim Berne.	BK	Neutral
It sounded as if the saxophonist had listened to a lot of Steve Coleman.	BK	Neutral
**Possible agreement: One performer "strongly disagrees" and the other "neutral" (N=1)**		
Between 3'50 and 3'56 the saxophone is a little bit out of tune.	MP	Negative
**Disagreement: One performer "agrees" and the other "disagrees" (N=43)**		
There was a certain sense of more sure-footedness after a minute.	MP	Positive
In the very beginning the saxophonist was slightly behind.	IP	Negative
The saxophone was very quiet and sort of polite.	MP	Positive
Around 0'47 the pianist is romanticizing things.	IP	Neutral
Between 0'14 and 1'01 the pianist is superimposing something over what the saxophonist is doing.	IP	Neutral
At 1'01 there is sort of a silence.	MP	Neutral
Around 1'25 there is kind of a cadence moment.	MP	Neutral
Right after 1'40 the performers go to the first section of flurries together.	IP	Neutral
Between 2'07 and 2'40 the performers are sitting pretty much in the same range.	IP	Neutral
Between 3'40 and 3'47 there is some kind of shimmering.	MP	Neutral
The music in this excerpt had more energy than the first excerpt.	MP	Positive
The music in this excerpt was more assertive than in the first excerpt.	MP	Positive
The saxophonist's scale and intervallic ideas in this excerpt were repetitive.	MP	Neutral
At 0'09 the saxophonist goes into repeated three notes.	IP	Neutral
At 1'07 it sounds as if the saxophonist is shifting to go into a certain space.	IP	Neutral
From 1'07 there is a little tension for the performers to find something together again.	IP	Positive
At 1'12 the performers are back to shimmering.	IP	Neutral
Between 1'39 and 1'51 the saxophonist is working with a major 7.	IP	Neutral
Around 1'50 the pianist is using low octaves as they did in the first excerpt.	IP	Neutral
Between 1'52 and 1'56 the saxophonist is phrasing.	IP	Neutral
Between 1'52 and 1'56 the pianist is playing almost like a bass player.	IP	Neutral
At 1'59 the music gets into another pulse.	MP	Neutral
Between 2'31 and 2'38 the pulse is "Stravinsky-esque."	MP	Neutral
Between 2'31 and 2'38 the pianist opens up the harmonies vertically.	IP	Neutral
Between 2'31 and 2'38 the pianist opens up a group of harmonies that then saxophonist can shoot in and out of.	IP	Positive

Between 3'13 and 3'17 what the pianist is doing is funny.	MP	Neutral
Between 3'13 and 3'18 what the pianist did is funny.	MP	Neutral
Between 3'22 and 3'34 both performers' lines are independent.	MP	Neutral
At 4'52 there is a cathartic moment from the saxophonist.	MP	Neutral
It sounds as if both performers were skipping through ideas and shapes.	IP	Neutral
It sounds as if the performers were both very conscious of each other's space.	IP	Positive
It sounds as if both performers tried to be maximalists and minimalists at the same time.	IP	Neutral
It sounds as if the saxophonist never loses sight of their instrument and what the instrument allows phrasing-wise as far as melodic possibilities.	IP	Positive
It sounds as if the performers' vocabularies overlapped but their processes are different.	BK	Neutral
The performers reached a kind of peak and then it came down.	IP	Neutral
Around 0'50 the saxophonist is trying to play chordally.	IP	Neutral
In this excerpt, the performers were spending more time exploring something, compared to the 1st excerpt.	IP	Positive
Between 0'00 and 0'15 after overblowing, the saxophonist goes to a minimalist style.	IP	Neutral
Between 0'00 and 0'42 the pianist is coming in with a harmonic idea that has some distance from what the saxophonist is doing.	IP	Neutral
Around 0'50 the saxophonist is playing a bit more with the sound.	IP	Neutral
Some of the intervallic language of the saxophonist is based on Messiaen.	MP	Neutral
It sounds as if the performers come out of the same influences.	BK	Neutral
It sounds as if the performers are from different circles of musicians.	BK	Neutral
**Disagreement: One performer "strongly agrees" and the other "disagrees" (N=5)**		
The performers strongly showed that they were hearing what the other person was doing.	IP	Positive
Between 4'31 and 4'42 the pianist really goes into an idiom.	IP	Neutral
Between 4'33 and 4'45 the performers are playing in a jazz pulse.	IP	Neutral
Between 1'56 and 2'00 the dialog is interesting because the performers are not being locked into a certain harmonic theme.	MP	Positive
Between 0'00 and 0'42 the performers are spending more time exploring something (compared to the first excerpt).	IP	Positive
**Disagreement: One performer "agrees" and the other "strongly disagrees" (N=2)**		
1'51 is the end of the major 7 gesture on the saxophone.	MP	Neutral
Between 0'33 and 1'01 the pianist is playing with arpeggiating, rippling patterns.	IP	Neutral
**Other: One performer selects "I don't understand" and the other "agrees" (N=4)**		
It sounded as if the performers played parallel lines so that they could each go off on different vectors.	IP	Neutral

Between 0'21 and 0'50 where they are playing the same ideas, it's just a couple of pitches that swirl around each other.	IP	Neutral
Between 3'13 and 3'18 what the pianist did is "show and end."	MP	Neutral
At 4'10 the saxophonist goes from repeating patterns into the pianist's rhythmic patterns.	IP	Neutral
**Other: One performer selects "I don't understand" and the other "neutral" (N=1)**		
It sounded as if the performers were playing parallel lines.	IP	Neutral
**Other: One performer selects "I don't understand" and the other "disagrees" (N=2)**		
It sounded as if they went easy with each other.	IP	Neutral
At 0'09 the pianist starts playing three notes that the pianist picks up on.	IP	Neutral
**Other: One performer selects "I don't understand" and the other "strongly disagrees" (N=1)**		
Between 2'07 and 2'40 there is a certain staticness.	MP	Neutral
